# A Pleiotropic Flowering Time QTL Exhibits Gene-by-Environment Interaction for Fitness in a Perennial Grass

**DOI:** 10.1093/molbev/msac203

**Published:** 2022-09-23

**Authors:** Xiaoyu Weng, Taslima Haque, Li Zhang, Samsad Razzaque, John T Lovell, Juan Diego Palacio-Mejía, Perla Duberney, John Lloyd-Reilley, Jason Bonnette, Thomas E Juenger

**Affiliations:** Department of Integrative Biology, University of Texas at Austin, Austin, TX, USA; Department of Integrative Biology, University of Texas at Austin, Austin, TX, USA; Department of Integrative Biology, University of Texas at Austin, Austin, TX, USA; Department of Integrative Biology, University of Texas at Austin, Austin, TX, USA; HudsonAlpha Institute for Biotechnology, Huntsville, AL, USA; Corporación Colombiana de Investigación Agropecuaria – AGROSAVIA, Centro de Investigación Tibaitatá. Kilómetro 14 vía Mosquera-Bogotá, Mosquera. Código postal 250047, Colombia; Kika de la Garza Plant Materials Center, USDA-NRCS, Kingsville, TX, USA; Kika de la Garza Plant Materials Center, USDA-NRCS, Kingsville, TX, USA; Department of Integrative Biology, University of Texas at Austin, Austin, TX, USA; Department of Integrative Biology, University of Texas at Austin, Austin, TX, USA

**Keywords:** *Panicum hallii*, flowering time, quantitative trait locus (QTL), gene-by-environment interaction, pleiotropy, local adaptation

## Abstract

Appropriate flowering time is a crucial adaptation impacting fitness in natural plant populations. Although the genetic basis of flowering variation has been extensively studied, its mechanisms in nonmodel organisms and its adaptive value in the field are still poorly understood. Here, we report new insights into the genetic basis of flowering time and its effect on fitness in *Panicum hallii*, a native perennial grass. Genetic mapping in populations derived from inland and coastal ecotypes identified flowering time quantitative trait loci (QTL) and many exhibited extensive QTL-by-environment interactions. Patterns of segregation within recombinant hybrids provide strong support for directional selection driving ecotypic divergence in flowering time. A major QTL on chromosome 5 (*q-FT5*) was detected in all experiments. Fine-mapping and expression studies identified a gene with orthology to a rice *FLOWERING LOCUS T-like 9* (*PhFTL9*) as the candidate underlying *q-FT5*. We used a reciprocal transplant experiment to test for local adaptation and the specific impact of *q-FT5* on performance. We did not observe local adaptation in terms of fitness tradeoffs when contrasting ecotypes in home versus away habitats. However, we observed that the coastal allele of *q-FT5* conferred a fitness advantage only in its local habitat but not at the inland site. Sequence analyses identified an excess of low-frequency polymorphisms at the *PhFTL9* promoter in the inland lineage, suggesting a role for either selection or population expansion on promoter evolution. Together, our findings demonstrate the genetic basis of flowering variation in a perennial grass and provide evidence for conditional neutrality underlying flowering time divergence.

## Introduction

Local adaptation is a key component of responses to changing environments and a central topic in modern evolutionary biology (Savolainen et al. 2013). Plants provide unique opportunities to study the ecological and evolutionary history of local adaptation, as they are unable to escape from danger and must tackle the challenges that local conditions present (Fournier-Level et al. 2011). The selection of favored phenotypes by local habitats usually leads to phenotypic differentiation among natural populations, thereby enhancing fitness and promoting local adaptation (Primack and Kang 1989; Sandring and Agren 2009; Larios and Venable 2018). One common hypothesis is that local adaptation driven by natural selection results in trade-offs involved in specialization (Kawecki and Ebert 2004; Mitchell-Olds et al. 2007). Alleles increasing fitness in one environment may through antagonistic pleiotropy result in decreases in fitness in other environments. Alternatively, local adaptation may arise through mutations that improve fitness locally, but that are neutral and generally have no fitness impact in other environments (i.e., conditional neutrality) (Fry 1996; Hall et al. 2010; Lowry et al. 2019). Determining the frequency of antagonistic pleiotropic versus conditionally neutral allelic effects is important for understanding the forces that maintain standing genetic variation, the importance of gene flow and recombination in facilitating or constraining adaptation, and the likelihood or extent of local adaptation in natural populations (Anderson et al. 2013). While progress has been made (Wadgymar et al. 2017), too few empirical studies have explored the genetic architecture of natural alleles and their interaction with native environments. Therefore, field studies bringing together the genetic basis and ecological significance of ecologically important traits are critically needed.

Reproductive timing is a key developmental decision in the life history for all species (Hall and Willis 2006; Verhoeven et al. 2008; Anderson, Lee, et al. 2011). In plants, depending on geography, the patterns of flowering time generally evolve in response to local environmental factors like the day-length, temperature, and different types of biotic or abiotic stresses through changes in flowering time pathway genes (Elzinga et al. 2007; Kim et al. 2009; Blackman 2017). As a complex trait, flowering time is often tightly integrated within the genetic networks of other traits related to plant growth and stress responses (Auge et al. 2019). Although it is not always clear whether this phenomenon is due to selection on flowering time itself or through other traits sharing the same genetic network, pleiotropy (one gene affecting multiple traits) is one of the most commonly observed attributes of genes involving flowering time pathways (Auge et al. 2019). For example, several main flowering time genes in *Arabidopsis thaliana* (*A. thaliana*), including *FLOWERING LOCUS T* (*FT*) and its regulators (e.g., *FRIGIDA* [*FRI*] and *FLOWERING LOCUS C* [*FLC*]), have shown pleiotropic effects on development (e.g., branching architecture and seed germination) and physiological (e.g., water use efficiency) characteristics (Chiang et al. 2009; Hiraoka et al. 2013; Lovell et al. 2013). In rice (*Oryza sativa*), several *FT* homologs (*HEADING DATE 3a* [*Hd3a*] and *RICE FLOWERING LOCUS T 1* [*RFT1*]) and their upstream regulators (*HEADING DATE 1* [*Hd1*] and *GRAIN NUMBER, PLANT HEIGHT AND HEADING DATE 7* [*Ghd7*]) have roles in vegetative and reproductive branching development (Xue et al. 2008; Zhang et al. 2012; Tsuji et al. 2015; Zhu et al. 2017). These results suggest potential adaptive roles of flowering time genes in the maintenance of diverse life-history strategies across different populations.

Determining the adaptive value at the gene level is critical to understand the molecular basis of local adaptation (Anderson, Willis, et al. 2011). To date, extensive inquiry into the genetics of adaptation have revealed quantitative trait loci (QTL) in many systems (Hall et al. 2010; Agren et al. 2013, 2017). These studies are especially potent in genetic models, such as *A. thaliana*, where the putative function of many genes can be well characterized (Agren et al. 2013, 2017). However, most such studies never test the adaptive value of individual genetic loci in the environments in which the traits evolved. In terms of the gene level, although more than 300 genes involved in flowering time regulation have been identified in *A. thaliana* (Bouche et al. 2016), only a few of them have been tested for fitness effects with mutants grown under realistic natural conditions (Scarcelli et al. 2007; Taylor et al. 2019). For nonmodel species, unfortunately, the challenge is more apparent because of the lack of genetic resources (e.g., mutants or genetic population) and related knowledge of the genetic architecture of target traits (e.g., QTL or candidate genes).

*P. hallii* is a genetically tractable native perennial grass occurring in North American with a geographic range that spans a number of ecoregions and native habitats (Lowry et al. 2012). Two major ecotypes of *P. hallii* are found in inland (*var. hallii*) or coastal (*var. filipes*) habitats across the southwest (Lowry et al. 2015; Palacio-Mejia et al. 2021). Consistent with differential adaptation to xeric inland and mesic coastal habitats, the HAL2 genotype, which is representative of *var. hallii* ecotype, flowers earlier and develops faster than the FIL2 genotype, representing the *var. filipes* ecotype (Lowry et al. 2015). Independent de novo genome assemblies and annotations have been built for the HAL2 and FIL2 accessions and F_2_ and recombinant inbred line mapping populations have been generated between these two genotypes (Lowry et al. 2015; Lovell et al. 2018; Khasanova et al. 2019). In this study, we leverage the resources of *P. hallii* to investigate the genetic basis of flowering time and test the adaptive value of a major genetic factor under field conditions. We are particularly interested in: 1) whether flowering time has evolved under natural selection in *P. hallii*? 2) What genomic regions contribute to flowering time divergence and genotype-by-environment (G × E) interactions between representative inland and coastal ecotypes? 3) Do the major flowering time QTL impact fitness through pleiotropy and display trade-offs or conditional neutrality under natural field conditions?

## Results

### Genotype-by-Environment Interactions and Natural Selection for Flowering Time

**Fig. 1.**
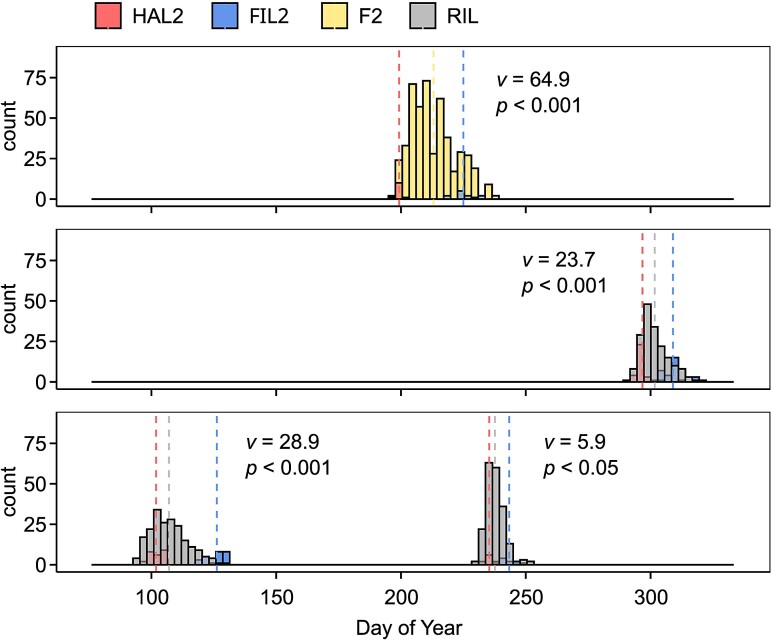
Flowering time variation of *Panicum hallii* in F_2_ and RIL populations. The distribution of flowering time in F_2_ population in 2014 (above), and RIL population in 2015 (middle) and 2016 (bottom) experiments. The parental (HAL2 and FIL2) and population (F_2_ and RIL) means are plotted above each distribution. The *v*-test results and *P*-values are presented for each population.

To probe the genetic architecture of evolved differences in flowering time between the ecotypes, we applied QTL mapping across different seasons using an F_2_ and recombinant inbred line (RIL) populations derived from HAL2 and FIL2. Despite substantial variation of photoperiod among the four controlled experiments in which flowering time was assayed, the HAL2 parent always flowered significantly earlier than the FIL2 parent (*t*-tests, *P* < 0.01 from all four experiments) (fig. 1 and supplementary table S1, Supplementary Material online). In the F_2_ population, the difference in mean flowering time between parents was 25.8 days (Cohen’s ds = 8.3), and this difference in three RIL populations ranged from 8.0 to 25.3 days (Cohen’s ds ranged from 3.2 to 7.4) (supplementary table S1, Supplementary Material online). The broad-sense heritability (*H*^2^) of flowering time in three RIL experiments in 2015 fall, 2016 spring, and 2016 summer were 0.42, 0.31, and 0.41, respectively. We observed positive genetic correlations less than a value of 1 across three seasonal RIL experiments (*r_g-fall-spring_* = 0.56, *r_g-fall-summer_* = 0.65, *r_g-spring-summer_* = 0.60), suggesting the presence of genotype-by-environment (G × E) at the trait level. We further tested for G × E across the RIL experiments using factorial linear models and detected a strong G × E interaction (*P* < 0.001) of flowering time over three seasons of study. These results show that flowering time variation between representatives of each ecotype was heritable and sensitive to different seasonal environments, potentially resulting from differential environmental sensitivity at discrete genetic loci. We used the Fraser *v*-test statistic based on segregating genetic variance to test for directional selection as an explanation for flowering time divergence. In all cases, the variances of parental flowering time values were greater than the variances among RILs and *v*-tests were highly significant (in all cases, *P* < 0.05) for flowering time measured in four experiments (supplementary table S2, Supplementary Material online). These results suggest historical directional selection underlying flowering time divergence among *P. hallii* ecotypes.

### Mapping QTL for Flowering Time in an F_2_ Population

**Fig. 2.**
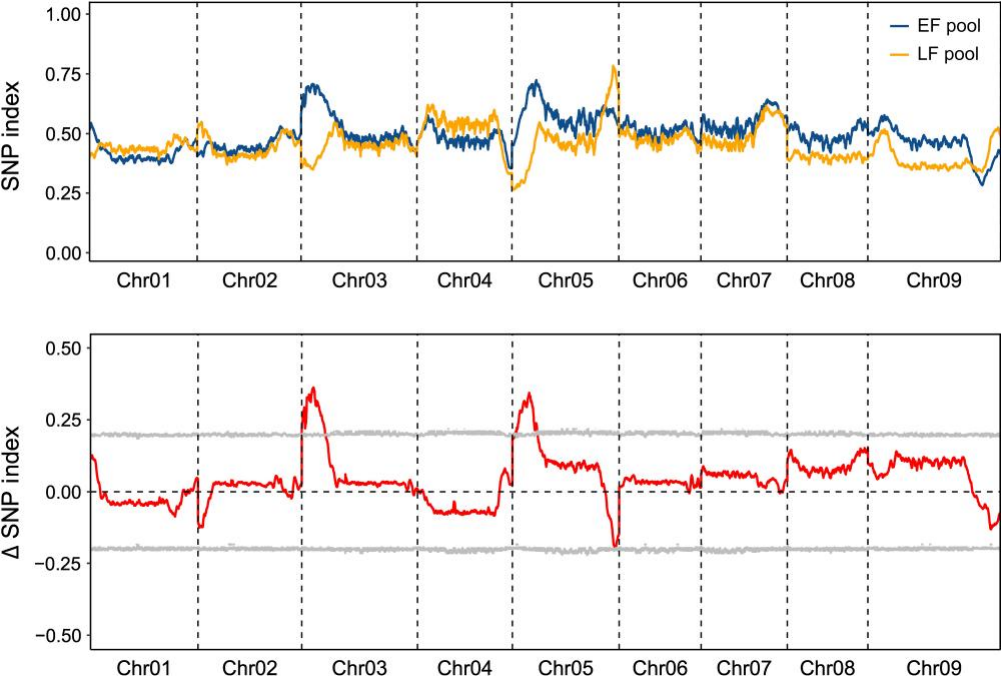
SNP index plots of early flowering (EF) and late flowering (LF) population and Δ (SNP index) plots generated by sliding-window analysis. The SNP index (ratio of the SNPs that are identical to those in the EF and LF pools), the Δ (SNP index) (subtracting the SNP index of the LF population from that of the EF population), and its 95% confidence interval are presented in the figure, respectively.

To investigate the genetic basis of flowering divergence between the ecotypes, we sequenced two bulk populations with extremely early or late flowering times (EF-pool and LF-pool), each consisting of ∼20% of the progeny from the tail of the F_2_ population. After mapping and single nucleotide polymorphism (SNP) calling, we explored allele frequency shifts in the bulked progeny by computed a SNP-index for the EF and LF pools as well as their differences, Δ (SNP-index). Two regions on chromosomes 3 and 5 had an average SNP-index higher than 0.7 in EF-pool and one region on chromosome 5 had an average SNP-index as low as 0.25 in LF-pool (fig. 2). By examining the Δ (SNP-index) plot, we identified two genomic regions exhibiting the highest Δ (SNP-index) values: the region on chromosome 3 from 0.3 to 9.9 Mb and the region on chromosome 5 from 0.5 to 11.1 Mb (95% confidence intervals) (fig. 2). To further confirm the flowering QTL detected by QTL-seq, we developed 13 insertion/deletion (InDel) markers in two regions (supplementary table S3, Supplementary Material online) and conducted a classical bi-parental QTL analysis within the same F_2_ population. We found the peak LOD scores at the marker M3-4979 on chromosome 3 (LOD = 15.3) and the marker M5-8935 on chromosome 5 (LOD = 12.1). Our results in the F_2_ population suggested that flowering time is controlled by a few major-effect QTL on chromosomes 3 and 5 in *P. hallii*.

### Mapping QTL for Flowering Time in a RIL Population

**Fig. 3.**
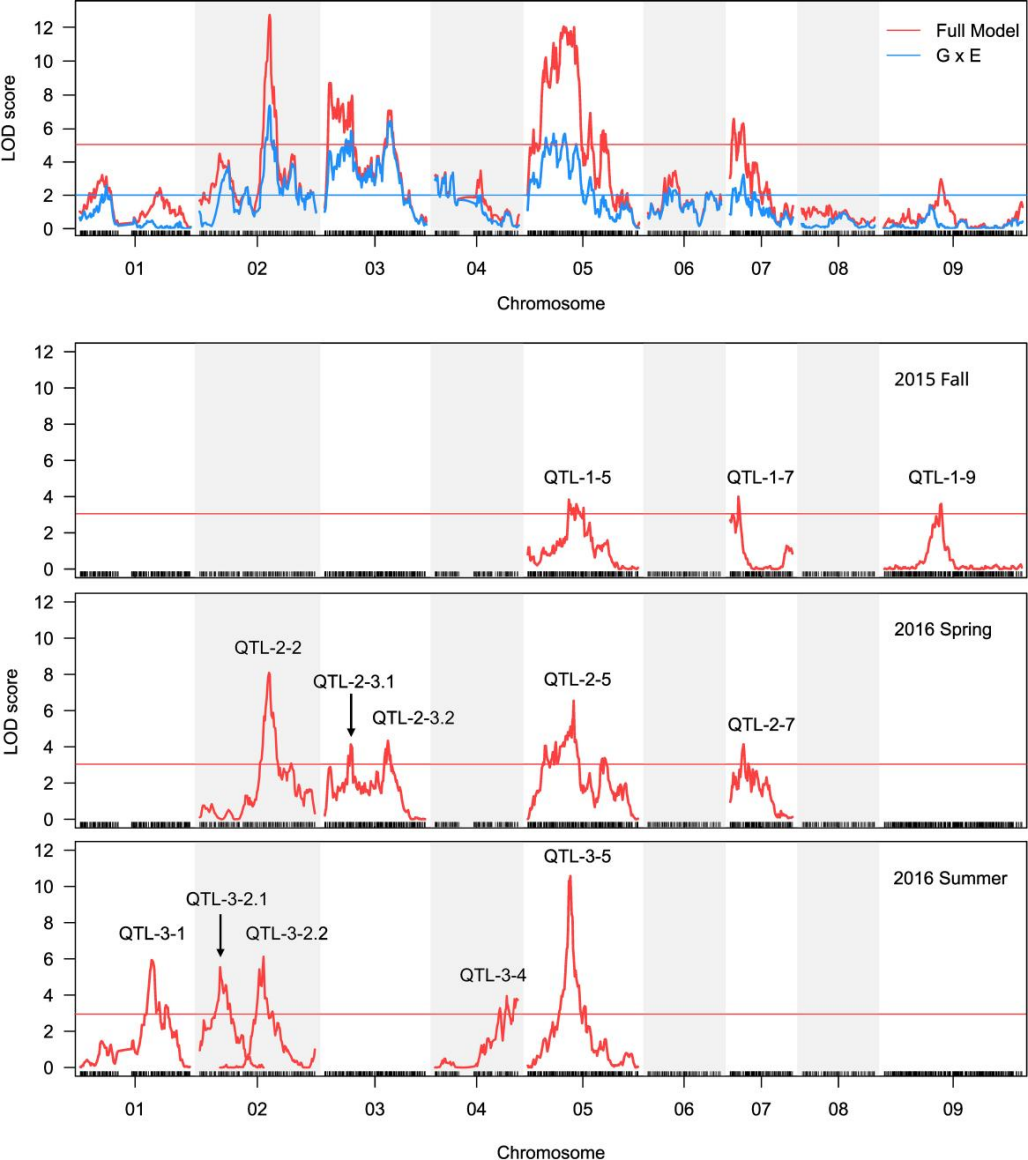
Location of flowering time QTL under different environmental conditions. QTL × E was tested as the difference between the full model including QTL × E interaction and a reduced additive model (Above). QTL mapping for each RIL experiment are shown below. The thresholds of significance are based on permutations. The name attributed to the different QTL are shown above the QTL peak.

To dissect genomic regions governing flowering differences and patterns of QTL-by-environment interaction (QTL × E), we mapped QTL in the RIL population grown across three experimental conditions using a modeling strategy incorporating QTL × E. QTL × E was tested as the difference between a full model incorporating seasonal environments as an interactive covariate versus a reduced model only controlling for additive effects of the environments. These analyses confirmed the major QTL on chromosomes 3 and 5 along with three novel QTL detected in the full model (fig. 3 and supplementary table S4, Supplementary Material online). Furthermore, by comparing the full model and the reduced model, we identified nine genomic regions exhibiting significant QTL × E, including all QTL detecting in the full model and four other regions on chromosomes 1, 2, 4, and 6 (fig. 3 and supplementary table S4, Supplementary Material online). The QTL effects showed that HAL alleles of all five QTL in the full model accelerated flowering in most seasons, except the QTL on the bottom of chromosome 3 in 2015 fall and 2016 summer (supplementary fig. S1, Supplementary Material online). Moreover, we observed stronger QTL effects for all five QTL in 2016 spring relative to the two other seasons (supplementary fig. S1, Supplementary Material online).

To further explore the genetic basis of flowering variation in each seasonal condition, we performed multiple QTL mapping based on stepwise modeling for each experiment separately. Here, we notate QTL by their season of occurrence and their chromosome location (QTL-season-chromosome). We detected three QTL in 2015 fall (QTL-1-5, QTL-1-7, and QTL-1-9), five QTL in 2016 spring (QTL-2-2, QTL-2-3.1, QTL-2-3.2, QTL-2-5, and QTL-2-7), and five QTL in 2016 summer (QTL-3-1, QTL-3-2.1, QTL-3-2.2, QTL-3-4, and QTL-3-5) (fig. 3 and supplementary table S5, Supplementary Material online). Most QTL had additive effects with FIL2 alleles delaying flowering, except two QTL (QTL-3-1 and QTL-3-2.1) detected in 2016 summer (supplementary table S5, Supplementary Material online). The proportion of total variation in flowering time explained by significant QTL in each experiment varied from 24.3% to 43.5% (supplementary table S5, Supplementary Material online). The additive effects of each QTL were 0.78–2.73 days and explained 5.9–17.8% of the phenotypic variation (supplementary table S5, Supplementary Material online). Among these, the QTL on chromosome 5 was identified across three seasons of study (QTL-1-5, QTL-2-5, and QTL-3-5) with the highest peak LOD scores during the summer of 2016 (QTL-3-5, LOD = 10.6) (fig. 3 and supplementary table S5, Supplementary Material online). Interestingly, we detected an epistatic interaction between QTL-3-5 and QTL-3-1 in 2016 summer (supplementary fig. S2, Supplementary Material online). Moreover, we detected three minor QTL (QTL-1-9, QTL-3-1, and QTL-3-4) that were not observed in our QTL × E analysis (fig. 3 and supplementary table S5, Supplementary Material online). These findings enhance our understanding of the genetic basis of flowering time and highlight the locus on chromosome 5 as a major flowering QTL in *P. hallii*.

### Fine Mapping of the Major Flowering Time QTL on Chromosome 5

**Fig. 4.**
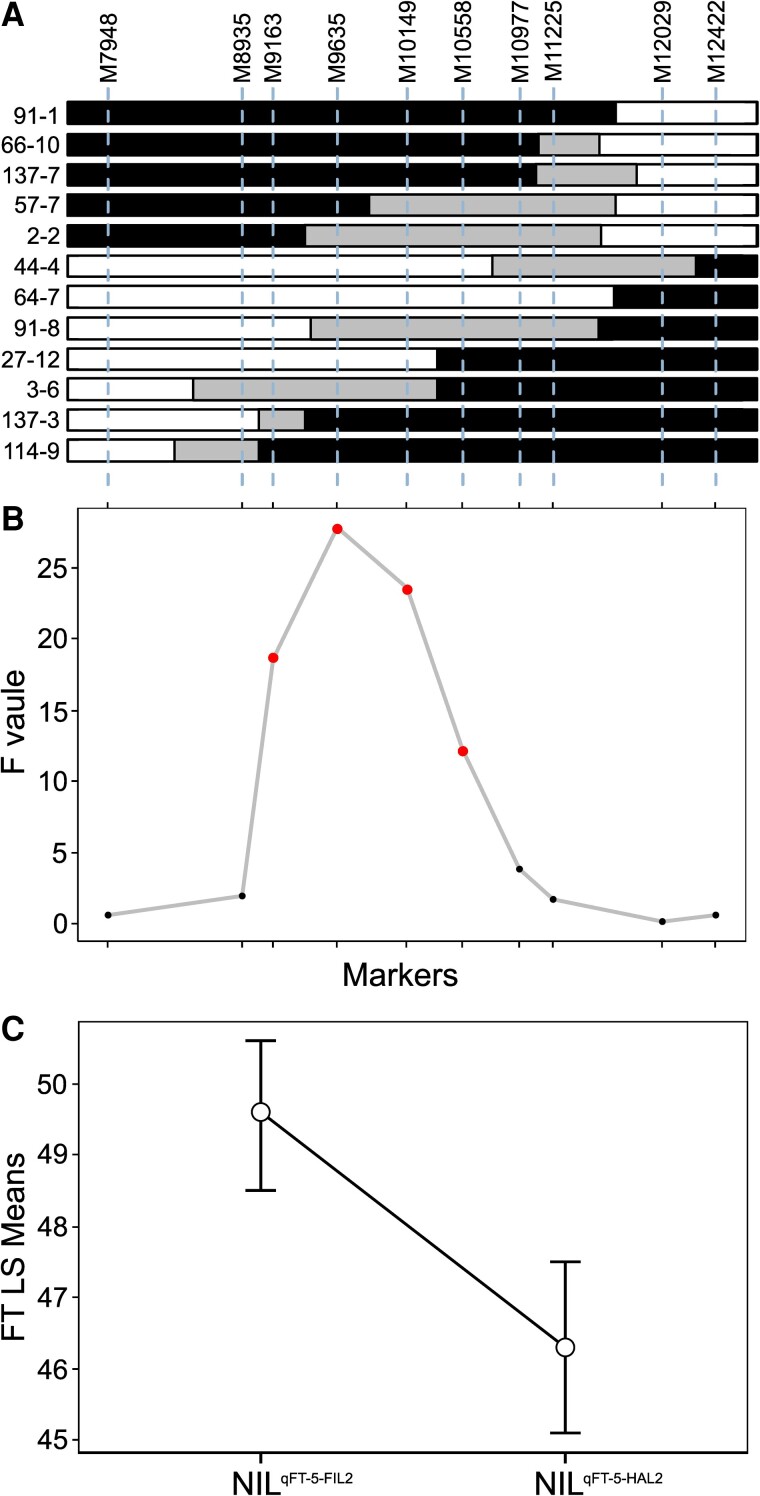
Fine-mapping of the major flowering time QTL, *qFT-5*. (*A*) The recombinants identified from HIF experiments. Black, white, and gray boxes indicate homozygous regions for HAL2, homozygous regions for FIL2, and heterozygous regions where recombination occurred, respectively. (*B*) The results of ANOVA examining the effect of genotypes across eight interval markers in recombinant-derived F_3_ families for flowering time. (*C*) The flowering time difference between NIL^qFT-5-FIL2^ and NIL^qFT-5-HAL2^ plants.

Given the importance of the QTL on chromosome 5 (*qFT-5*), we used a heterogeneous inbred family (HIF) strategy for fine-mapping (Tuinstra et al. 1997). A RIL line (FH-312) that was heterozygous in the *qFT-5* region was chosen to develop our HIF (supplementary fig. S3, Supplementary Material online). First, we observed that the HIF progenies carrying FIL2 homozygous alleles at *qFT-5* region displayed a significantly later flowering time than those carrying HAL2 homozygous alleles (*t*-test, *P* < 0.01; supplementary fig. S4, Supplementary Material online), suggesting that the causal region of *qFT-5* is located between two flanking markers. To refine the physical interval, we identified 12 recombinants among the progeny using two flanking markers M5-7948 and M5-12422 (fig. 4*A*) and conducted QTL mapping in a recombinant-derived F_3_ population. We detected significant flowering time differences from marker M5-9163 to M5-10558 with the peak at marker M5-9635 (*F* = 27.775, *P* < 0.001; lsmeans-FIL2 = 48.1 ± 0.4; lsmeans-HAL2 = 45.2 ± 0.4; contrast estimate FIL2-HAL2 = 2.9 ± 0.5, *P* < 0.001) (fig. 4*B*). It is worth noting that the physical position of marker M5-9635 (9,038,694–9,039,322 bp) is only ∼30-kb away from the QTL LOD peak (9,068,928 bp) identified in RIL mapping. Accordingly, we created a pair of nearly-isogenic lines (NIL) (NIL^qFT-5-FIL2^ and NIL^qFT-5-HAL2^) spanning a 380-kb region around the peak region. We detected a significant flowering time difference between NIL^qFT-5-FIL2^ and NIL^qFT-5-HAL2^ plants (lsmeans-NIL^qFT-5-FIL2^ = 49.6 ± 0.4; lsmeans-NIL^qFT-5-HAL2^ = 46.3 ± 0.5; contrast estimate NIL^qFT-5-FIL2^–NIL^qFT-5-HAL2^ = 3.3 ± 0.7, *P* < 0.001) (fig. 4*C*), suggesting that the 380-kb region may harbor the candidate gene for *qFT-5*. We identified 60 and 62 annotated genes in HAL2 and FIL2 genome assemblies, respectively (supplementary table S6, Supplementary Material online). There is no functional annotation for two presence/absence genes, and *K_a_*/*K_s_* estimation supports purifying selection as the major driver of protein evolution for genes in the QTL interval (supplementary table S6, Supplementary Material online). We examined the expression variation of candidate genes in the interval between HAL2 and FIL2 from an existing high replicated transcriptome dataset (unpublished data, see Materials and Methods). This analysis led us to identify 16 differentially expressed genes (*P* < 0.01), including a phosphatidylethanolamine-binding protein homologous to the rice *FT-like 9* gene (*PhFTL9*) (supplementary table S6, Supplementary Material online). We further performed qPCR analysis and confirmed that the expression of *PhFTL9* in HAL2 plants was significantly higher than that in the FIL2 plants (supplementary fig. S5, Supplementary Material online). In plants, members of the *FT* and *FT-like* gene family are florigens and their gene expression variability has been shown to impact flowering time (Komiya et al. 2008; Turck et al. 2008; Schwartz et al. 2009). Therefore, we hypothesize that *PhFTL9* is a likely candidate gene underlying *q-FT5*.

### The *qFT-5* Conditionally Affects Fitness-Based Adaptation in Native Fields

**Fig. 5.**
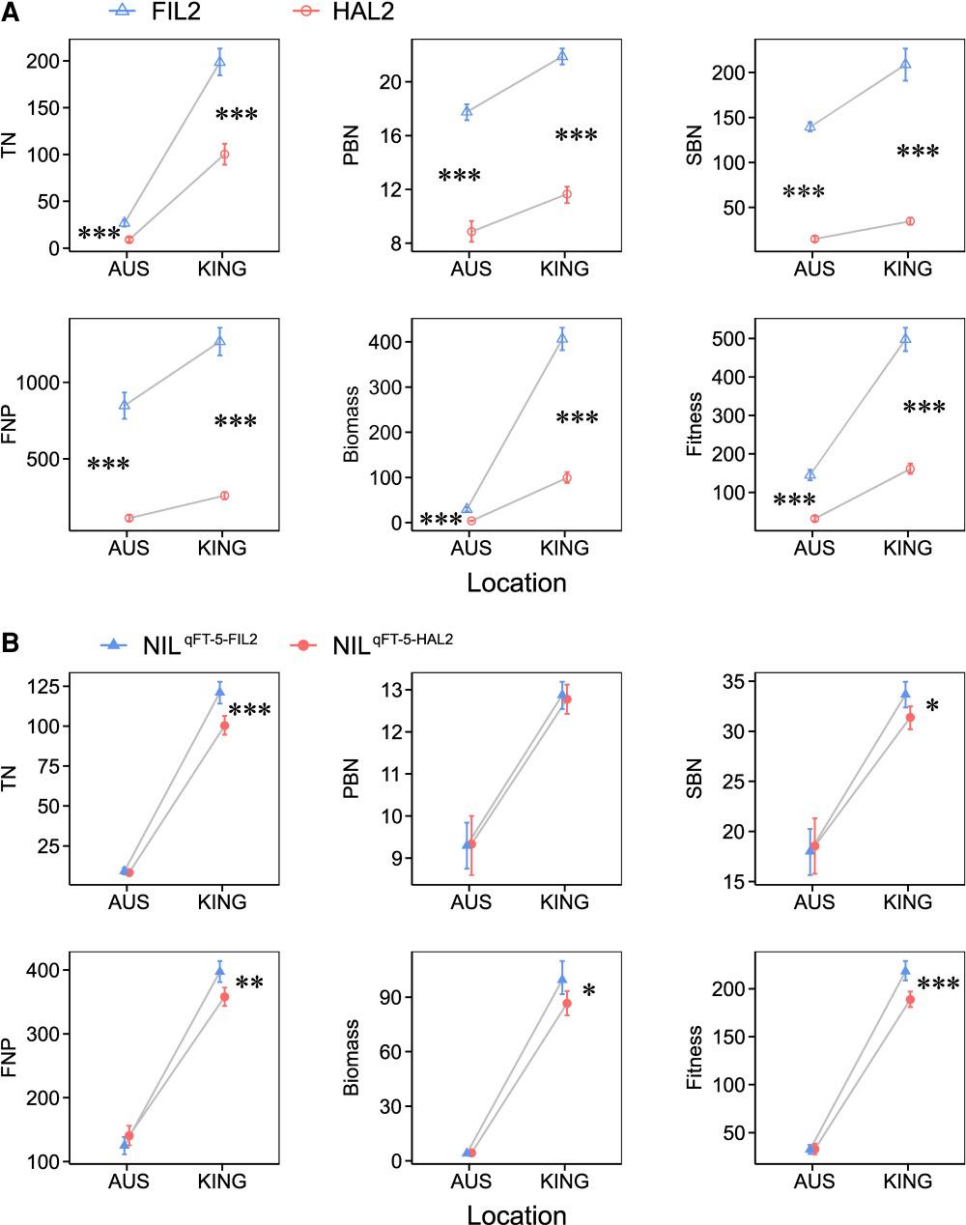
Fitness-related traits of parents (*A*) and HIF plants (*B*) in reciprocal transplant experiments. The *x*-axis indicates two field locations for the reciprocal transplanting. AUS indicates Austin and KING indicates Kingsville. The *y*-axis indicates fitness-related traits for the measurement. The results of two-way ANOVA examining the effect of location and genotype; contrasts were used to test the effect of genotype separately by site. *P < 0.05; **P < 0.01; ***P < 0.001. TN, tiller number, PBN, primary branch number, SBN, secondary branch number, FNP, flower number per panicle.

To investigate the ecological and adaptive significance of *qFT-5*, we performed a field reciprocal transplant experiment using NIL pairs (NIL^qFT-5-FIL2^ and NIL^qFT-5-HAL2^) and two parents (FIL2 and HAL2) grown near their location of collection, Kingsville and Austin, TX, respectively. We measured performance traits related to growth and fitness over the course of a single summer growing season. For both parents, all fitness-related traits were significantly higher at the coastal site than at the inland environment (fig. 5). The location × genotype interaction for all fitness-related traits was statistically significant in the comparison of parental transplants with a two-way ANOVA test, especially for above-ground biomass per plant (*F* = 357.5, *P* < 0.001) and number of seeds per plant (*F* = 110.1, *P* < 0.001) (fig. 5 and supplementary table S7, Supplementary Material online). FIL2 outperformed the HAL2 parent for biomass and fitness at both locations, but to an increased degree at the coastal site. This pattern is in conflict with a hypothesis of local adaptation over this summer study season. For NIL pairs, we observed a significant location × genotype interaction for most fitness-related traits except the number of primary and secondary branches per panicle (supplementary table S7, Supplementary Material online). In comparisons between NIL pairs, NIL^qFT-5-FIL2^ plants produced more tillers, biomass, and seeds relative to NIL^qFT-5-HAL2^ plants at Kingsville (number of tillers per plant mean, NIL^qFT-5-FIL2^ = 121.1 ± 3.6, NIL^qFT-5-HAL2^ = 100.3 ± 2.9, *P* < 0.001; number of secondary branches per panicle mean, NIL^qFT-5-FIL2^ = 33.7 ± 0.7, NIL^qFT-5-HAL2^ = 31.4 ± 0.6, *P* < 0.01; number of flowers per panicle mean, NIL^qFT-5-FIL2^ = 397.1 ± 8.2, NIL^qFT-5-HAL2^ = 357.9 ± 7.7, *P* < 0.001; above ground biomass per plant mean, NIL^qFT-5-FIL2^ = 99.4 ± 4.6, NIL^qFT-5-HAL2^ = 86.6 ± 3.5, *P* < 0.05; number of seeds per plant mean, square root transformation, NIL^qFT-5-FIL2^ = 218.1 ± 5.0, NIL^qFT-5-HAL2^ = 188.7 ± 4.2, *P* < 0.001) (fig. 5), suggesting a fitness advantage of the local allele at *qFT-5* at the coastal environment during our season of study. In contrast, no statistically significant differences were recorded in fitness-related traits at the inland environment, Austin (fig. 5). These results suggested that the major flowering time QTL, *qFT-5*, conditionally controls fitness-based adaptation in *P. hallii*.

### Molecular Evolution of *FTL9* in Panicoid Grasses

*FT* family genes have been reported as selection targets in multiple species (Blackman et al. 2010; Guo et al. 2018; Wang et al. 2018). Therefore, we investigated sequence evolution in the coding and promoter region of *FTL9* across panicoid grasses and within *P. hallii*. In panicoid grasses, *K_a_*/*K_s_* ratio tests of neutrality showed that all orthologue pairs have a ratio varying from 0.066 to 0.457 (*P* < 0.01) (supplementary table S8, Supplementary Material online), suggesting that *FTL9* has generally undergone purifying selection. In *P. hallii*, we only found two synonymous changes and a single amino acid substitution (S2L) between HAL2 and FIL2 (supplementary fig. S6, Supplementary Material online). This amino acid substitution is not involved in the known functional regions of the protein, like the potential ligand-binding pocket or the external loop domain (Ho and Weigel 2014), suggesting that the function of *PhFTL9* is likely conserved between the two genotypes.

**Fig. 6.**
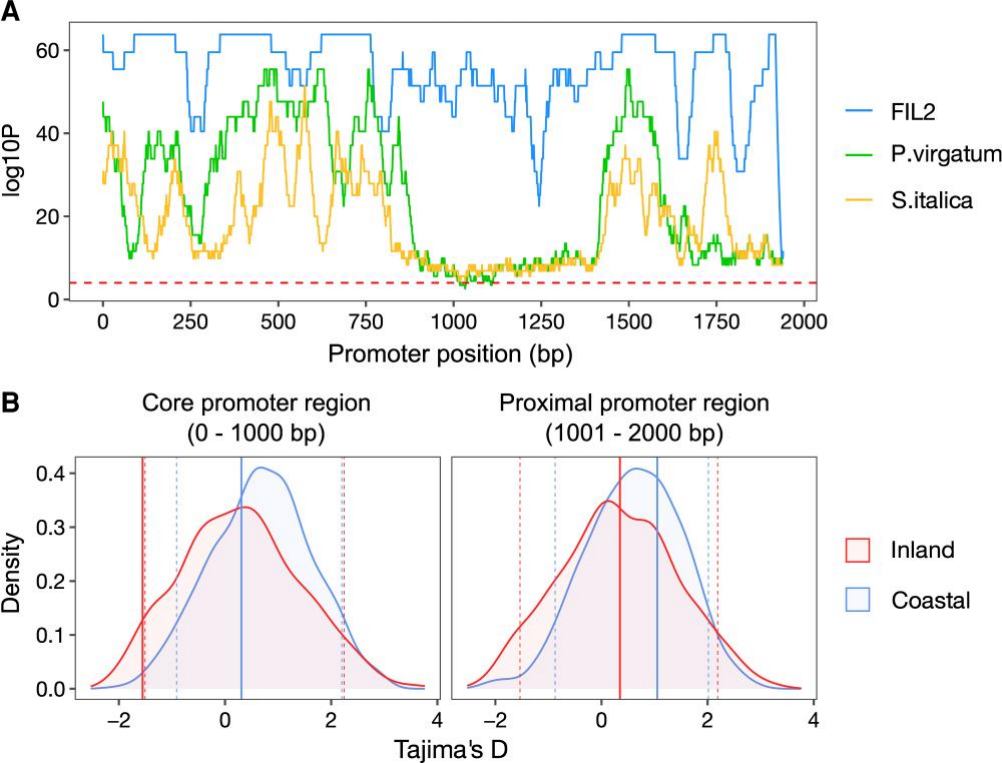
Sequence variation of *PhFTL9* promoter in *P. hallii* and related panicoid grasses. (*A*) Evolutionary conserved regulatory regions of *PhFTL9* homologs in panicoid grasses. The 2-kb promoter sequences from *P. hallii* HAL2 genotype are used as the reference and window size is setting as 60-bp. The *y-axis* gives the log10 transformed *P*-value obtained by aligning one window at this position to any window in *P. hallii* FIL2, switchgrass (*P. virgatum*), and *S. italica*. The dashed line indicates the raw score corresponding to the significance threshold for *P* = 0.00001. (*B*) The distribution of Tajima's D from the core (left) and proximal (right) promoter regions between inland and coastal groups. The values of Tajima's D from *PhFTL9* promoters are marked by solid lines. 5% and 95% thresholds were determined from 1000 random core/proximal promoters and were indicated by dashed lines.

We then used phylogenetic footprinting of the 2 kb promoter among *FTL9* orthologues in panicoid grasses to characterize sequence conservation. We observed a broad conserved block at the core promoter region (0–0.9 kb) and a small conserved block at the proximal promoter region (1.4–1.8 kb) (fig. 6*A*), implicating these conserved regions in gene regulation of *FTL9* in panicoid grasses. Furthermore, we measured Tajima’s D and nucleotide diversity (Pi) at the promoter of *PhFTL9* using genomic sequences from a *P. hallii* diversity panel previously subjected to high-throughput sequencing, including 49 inland (*var. hallii*) and 10 coastal (*var. filipes*) accessions, and compared them with 1,000 randomly chosen core promoters (see Materials and Methods for details). Intriguingly, only the core promoter region (0–1 kb) of *FTL9* from the inland group was detected as a significant outlier (threshold quantile = 5%) (the values of Tajima’s D: FTL9-D_inland-core_ = –1.555, FTL9-D_inland-proximal_ = 0.347; FTL9-D_coastal-core_ = 0.307, FTL9-D_coastal-proximal_ = 1.051) (fig. 6*B*). Similarly, the inland group also exhibited a considerably low nucleotide diversity for the core promoter region (0–1 kb) of *FTL9* compared with 1,000 randomly chosen core promoters (the values of Pi: FTL9-Pi_inland-core_ = 0.00024, FTL9-Pi_inland-proximal_ = 0.00182; FTL9-Pi_coastal-core_ = 0.00614, FTL9-Pi_coastal-proximal_ = 0.00598) (supplementary fig. S7, Supplementary Material online). These patterns signified an excess of low frequency polymorphisms at the core promoter region of *FTL9* relative to expectation, possibly related to population expansion after a bottleneck or a selective sweep at this locus.

## Discussion

### The Genetic Architecture of Flowering Time in *P. hallii*

*A. thaliana* and rice have served as models to understand the mechanisms of flowering time regulation in annual plants (Hayama and Coupland 2004; Izawa 2007). A core regulator involved in the molecular network of flowering is the *A. thaliana* gene *CONSTANS* (*CO*) and its rice orthologue *HEADING DATE 1* (*Hd1*), encoding zinc-finger transcriptional factors with the *CO*, *CO-like*, and *TOC1* domains (Putterill et al. 1995; Yano et al. 2000). A monocot specific transcriptional activator *EARLY HEADING DATE 1* (*Ehd1*), encoding a B-type response regulator, integrates various molecular signals and induces flowering in short days (Doi et al. 2004; Galbiati et al. 2016). These upstream regulators integrative environmental signaling and drive the florigens *FT* and *FT-like* genes to trigger properly timed flowering (Doi et al. 2004; Tiwari et al. 2010). These advances have facilitated our understanding and insight into mechanisms controlling flowering in *A. thaliana* and in crop domestication.

As a native perennial grass, *P. hallii* germinates and flowers in both spring and fall through the integration of complex seasonal and environmental signals associated with local conditions. Therefore, the genetic basis of flowering time and its potential molecular mechanisms in *P. hallii* could be unique relative to our understanding of flowering time in most annual monocarpic species. Our previous *P. hallii* studies identified two flowering time QTL in an F_2_ population and two panicle emergence QTL in a RIL population with a QTL on chromosome 5 (*qFT-5* in this study) found in both experiments (Lowry et al. 2015; Khasanova et al. 2019). In this study, several major QTL were identified in the F_2_ or RIL population under a given environment, indicating that flowering time variation between HAL2 and FIL2 is impacted by a relatively small number of large effect QTL. This is similar to the results of many native species (e.g., *A. thaliana*, *Brachypodium distachyon*, and monkeyflower [*Mimulus guttatus*]), where flowering variation is often impacted by a few loci with major effects (Salome et al. 2011; Fishman et al. 2014; Woods et al. 2017). Among the major QTL we discovered, *qFT-5* was uniformly detected across different experiments with considerable QTL × E interactions across different environments, signifying an essential role of *qFT-5* in flowering variation in *P. hallii*. Epistatic interaction was detected between the *qFT-5* region and other chromosome regions, suggesting its important role in flowering regulation networks. Combining the results from fine-mapping and differential expression analysis, we narrowed the *qFT-5* interval to 16 annotated genes in a 380-kb region. There are no genes known to impact flowering in this interval except an orthologue of *FT-like 9* (*FTL9*), suggesting it is a good candidate for *q-FT5*. Although it is well known that the *FT* gene family is involved in the regulation of flowering time, most studies focus on *FT* and its orthologues (e.g., *Hd3a* and *RFT1* in rice) as the florigen promoting flowering (Komiya et al. 2008; Schwartz et al. 2009). *FTL9* has not been determined as a cause of natural variation in flowering time in rice, the classic monocot model system. However, this novel *FT* family member has been strongly associated with flowering time in natural populations of switchgrass (Grabowski et al. 2017), a close relative of *P. hallii*. Moreover, a recent study suggested that natural variation of *FTL9* conferred competence to flower under short-day vernalization in *B. distachyon* (Woods et al. 2019). Additionally, *FTL9* orthologs in typical short-day crops (e.g., *CENTRORADIALIS 12* [*CN12*] in *Zea mays* and *Sorghum bicolor*) were shown to behave as floral activators with photoperiod sensitivity dependence (Meng et al. 2011; Mullet *et al*. 2011; Wolabu et al. 2016). These results suggest that *FTL9* could be a good candidate gene involved in flowering time variation and gene-by-environment interaction for fitness in temperate grasses. Further molecular evidence (e.g., CRISPR-cas9) will be needed to prove the function of *FTL9* underlying *q-FT5* in *P. hallii*.

We also detected a number of small effect QTL that have not been identified before in our earlier mapping studies. Most of them were only identified in specific environments. For example, QTL-1-7/QTL-2-7 and QTL-1-9 are specifically detected under spring or fall short-day conditions. The mapping interval of QTL-1-7/QTL-2-7 and QTL-1-9 spans several important photoperiodic flowering time genes including *Ghd7* and *Ehd1* (Doi et al. 2004; Xue et al. 2008). *Ghd7* is the ortholog of *VERNALIZATION2* (*VRN2*) in temperate grasses, including *B. distachyon* and wheat (*Triticum aestivum*) (Turner et al. 2013; Woods et al. 2019). *GHD7/VRN2* is expressed in long- but not short-day conditions and represses the expression of *FTL9* in *B. distachyon* and the expression of *Ehd1* in rice (Xue et al. 2008; Woods et al. 2019). Although we did not detect an interaction between QTL-1-7/QTL-2-7 and QTL-1-9, transcriptome data supports the presence of the *Ghd7-Ehd1* regulatory module in *P. hallii* (Weng et al. 2019). In addition, QTL-3-2.1 and QTL-3-4 are only detected in RIL population in 2016 summer. We observed orthologues of rice *Ghd7.1*/*OsPRR37* and *Hd3a* (*FTL2*) located in the mapping interval of QTL-3-2.1 and QTL-3-4, respectively. In rice, *Ghd7.1*/*OsPRR37* regulates flowering time and plays an important role in a wide range of adaptation (Koo et al. 2013; Yan et al. 2013), while *Hd3a* is an orthologue of *A. thaliana FT* gene, which is a systemically mobile signal to initiate flowering (Kojima et al. 2002; Tamaki et al. 2007). Further fine mapping and studies on the mechanism of these QTL will provide a unique opportunity to understand the genetic architecture of flowering time in perennial temperate grasses.

### Pleiotropy of Flowering Time Genes and its Interaction With Complex Environments

Pleiotropy is defined as one gene affecting multiple traits. This phenomenon is widely observed for genes associated with the flowering time pathway (Auge et al. 2019). In our study, we observed broad pleiotropic effects of *qFT-5* on growth and fitness-related traits including tiller number, panicle branching number, total flower number and biomass under natural field conditions. Our fine-mapping results and supporting evidence point to *FTL9* as the causal gene underlying these pleiotropic effects. As the integrator of flowering signal networks, it is perhaps unsurprising that *FT* genes often have pleiotropic effects on the coordination of growth and development in addition to flowering. For example, two *FT* family genes, *FT* and *TSF*, were shown to interact with *BRC1*, a *TB1* clade gene, to modulate lateral shoot outgrowth and repress the floral transition of the axillary buds in *A. thaliana* (Niwa et al. 2013). Similarly, rice florigen genes, *Hd3a* (*FTL2*) and *RFT1* (*FTL3*), modulates lateral branching and influences yield-related traits under laboratory or agronomic environments (Tsuji et al. 2015; Zhu et al. 2017). These results suggest that the pleiotropy of flowering time genes is broadly evolutionary conserved.

More interestingly, we found that the pleiotropy of *qFT-5* locus is environmentally dependent in our field reciprocal transplant experiment. We only observed pleiotropic phenotypes in the mesic habitat of Kingsville TX but not in the more xeric Austin TX location. A similar environment-dependent pleiotropic effect was reported in the flowering time gene *Ghd7*, which is an upstream regulator of *FT* family genes in rice (Weng et al. 2014). As a photoperiodic gene, *Ghd7* responses to different environmental stress signals, including ABA and drought (Weng et al. 2014). Moreover, it has been reported that the *FT* family genes, *Hd3a* and *RFT1*, integrate photoperiodic and drought stress signals to control flowering time in rice (Galbiati et al. 2016). Therefore, one explanation for the environment-dependent pleiotropy of flowering time genes is that these genes differentially respond to complex and often synergistic environmental signals, especially through the integration of signals from light, temperature, and different abiotic stresses. Our recent transcriptome profiling in *P. hallii* has demonstrated that *PhFTL9* expression is photoperiodic (Weng et al. 2019), but day-length is not the likely driver of the differences we observed between sites given the similar photoperiods of these locations over the course of our experiments. Interestingly, the expression of *PhFTL9* was regulated by a drought treatment in a previous *P. hallii* field experiment (Lovell et al. 2016). Therefore, it is plausible that *PhFTL9* differentially integrates day-length and drought stress signals in *P. hallii* to coordinate flowering time and other development processes at different locations. This type of process may explain the conditional nature of pleiotropy of *qFT-5* as well as its fitness effect. Additionally, research in *B. distachyon* has highlighted the antagonistic role of the *FTL9* orthologue in flowering regulation under different day-length conditions (Qin et al. 2019). These results suggest that *FTL9* plays a multifaceted role in the fine-tuning and modulation of photoperiodic flowering in temperate grasses. Further study of the molecular function and the dynamic expression of key flowering time genes across different representative natural habitats will improve the understanding of their roles in environment-induced pleiotropy.

### Contribution of Flowering Time Variation in Local Adaptation

Flowering time often experiences strong selection within and among natural populations and appropriate phenology is widely considered to be critical for successful reproduction and local adaptation (Anderson, Willis, et al. 2011). Here, we used a test based on segregating trait variance (Fraser 2020) and a reciprocal common garden study to evaluate the impact of selection on flowering time. The observed pattern of QTL effects and the pattern of trait genetic variance in our crosses are consistent with historical directional selection driving the divergence of flowering time between the upland and lowland ecotypes. In contrast, we did not observe a reciprocal home site advantage from our common garden studies, suggesting that the upland and lowland ecotypes did not exhibit fitness tradeoffs during our specific study season. While it is common for local genotypes to outperform foreign genotypes in common garden studies (Kawecki and Ebert 2004; Leimu and Fischer 2008), the degree of local adaptation observed is likely to depend on the vagaries of the period of study in ecological time, especially for long lived perennial. For example, we have recently seen evidence of strong local adaptation between upland and lowland ecotypes in this system related to early seedling recruitment (Razzaque and Juenger 2021). It is possible that fitness related tradeoffs would also be observed at the adult stage if we were to expand to a better estimate of lifetime fitness measured over successive growing seasons or across periods that experience more biotic or abiotic stress. Future field studies with expanded sampling of environments, genetic diversity, and spanning longer ecological time will be valuable complements to our current study of *P. hallii*.

Increasing evidence suggests that the genetic underpinnings of local adaptation are commonly explained by conditionally neutral loci (Lowry et al. 2019). To date, most flowering time variation has been found in coding regions. However, the gain or loss of regulatory motifs may also be critical to the accurate sensing of cues and appropriate responses driving the shift from vegetative to reproduction and local adaptation. For example, natural variation of *FT* in *A. thaliana* is associated with expression differences controlled by variants at *cis*-regulatory sequences, including small sequence polymorphisms (SNP or InDel) or large structural variations (Schwartz et al. 2009; Liu et al. 2014). In crop plants, a SNP in the promoter of *ZCN8*, a florigen gene in maize (*Z. mays*), shows a strong association with flowering time and with differential binding by its upstream flowering activator (Guo et al. 2018). In term of the local adaptation, expression polymorphisms at environmentally sensitive promoter elements may be a common mechanism of conditional neutrality. This hypothesis has been specifically supported in *A. thaliana* as fitness advantage underlying flowering time mutants is only observed in specific environments. A good example is that drought may modulate the effect of expression variation at *FRI*, with the functional allele promoting the elevated expression of its downstream gene *FLC* in drier environmental conditions (Lovell et al. 2013; Lowry et al. 2013).

Here, our study demonstrated conditional neutrality of fitness effects at the *qFT-5* QTL, corresponding to the known biological role of the *FTL9* gene, and likely driven by environmentally sensitive promoter divergence. Interestingly, our previous study reported a trans-by-drought treatment interaction for the abundance of *PhFTL9* transcripts in a drought experiment (Lovell et al. 2016), suggesting that the expression of *PhFTL9* is regulated by a trans QTL interacting with drought stress. In this study, we identified significantly low Tajima’s D and Pi in the core promoter region of *PhFTL9* from the inland population, suggesting an excess of low-frequency polymorphisms and low nucleotide diversity relative to the background expectation. These patterns suggested that the core promoter region of *PhFTL9* could be a target for either the action of selection (purifying or positive/sweep) or impacted by population expansion after a bottleneck event. The gain/loss of particular motifs in this region, for example, CCAAT binding sites (Tiwari et al. 2010), could be important for adaptation during the movement of *P. hallii* from eastern coastal regions into the inland deserts. We see no evidence of intermediate frequency alleles in either the coastal or inland lineage, as might be anticipated under a model of spatially or temporally variable selection. However, we emphasize that it is unclear what type of population genetic signature to expect in the case of dynamic and spatially variable selection, especially in the case of transient conditionally neutral alleles. Several simulation studies have suggested conditionally neutral alleles may be more challenging to detect from genome scans or traditional tests of non-neutral evolution (Tiffin and Ross-Ibarra 2014; Mee and Yeaman 2019; Yeaman 2022). Importantly, our observation of conditional neutrality may be limited to the specific season or environments of our study. How often do flowering time or pleiotropic loci exhibit beneficial, neutral, or deleterious effects? How variable are the fitness impacts of *qFT-5* alleles across spatial variation in habitats, temporal patterns across seasons, or over the span of fitness accrued across the lifetime of a perennial individual? Our study is one of the few showing how individual flowering time QTL contributes to local adaptation in the field. Future studies based on additional field work will helpful clarify the ecological drivers of *qFT-5* fitness effects and will improve our understanding of the molecular underpinnings of adaptation and the evolution of development through pleiotropic effects.

## Materials and Methods

### Plant Materials, Growth Conditions, and Phenotyping in Greenhouse Experiments

*P. hallii* F_2_ and RIL population were generated from a cross between two natural inbred accessions HAL2 (*var. hallii*) and FIL2 (*var. filipes*) (Lowry et al. 2015; Khasanova et al. 2019). HAL2 is an accession collected at the Lady Bird Johnson Wildflower Center in Austin, TX (30.19°N, 97.87°W), which is a seasonally xeric savanna habitat. FIL2 is an accession collected near the Corpus Christi Botanical Gardens in South Texas (27.65°N, 97.40°W), which has mesic coastal prairie habitat. A new F_2_ population of 493 individuals was developed for bulked segregant analysis in this study. A subset (212) of the available RIL lines were employed for genetic mapping studies (Khasanova et al. 2019). Seeds of F_2_ and RIL lines were scarified by sandpaper and placed on moistened sand in Petri dishes with a 12 h-light and 12 h-dark photoperiod at 25°C in the growth chamber. Five days after germination, individual seedlings were transplanted into Promix:Turface:Profile mixture (6:1:1) in square 8.9 cm plastic pots and grown with a randomized design on the bench of the greenhouse at the Brackenridge Field Laboratory at the University of Texas at Austin in Austin, TX. The germination date of F_2_ population was June 15 (early summer) in 2014 and the average day-length during the F_2_ experiment was 13.7 h. The germination date of three RIL experiments were September 5 (fall) in 2015, March 3 (spring) in 2016, and July 12 (late summer) in 2016. Flowering time was recorded as the number of days from germination to the day of first panicle emergence from the leaf sheath. Three to eight replicates from each RIL lines were measured for flowering time depending on space and plant availability. Day-length was calculated as a function of latitude and day of year for the experimental location as described before (Forsythe et al. 1995). The average day-length during the 2015 fall, 2016 spring, and 2016 late summer RIL experiments was 11.6, 12.6, and 13.3 h, respectively, obtained from the germination date to the date of the last flowering plant. The day/night temperature was controlled at 28°C/25°C in the greenhouse for all experiments.

### Generation and Analysis of QTL-seq in F_2_ Population

For QTL-seq, two DNA pools, including an early flowering pool (EF-pool) and late flowering pool (LF-pool) were constructed, respectively, by mixing an equal amount of DNA from 100 early flowering (31–38 days to flower) and 100 late flowering (54–68 days to flower) F_2_ plants from the 2014 summer experiment. Pair-end sequencing libraries (read length 150 bp) were prepared by Genomic Sequencing and Analysis Facility in the University of Texas at Austin with an Illumina HiSeq 2500 platform according to the standard manufacturer’s instructions. Quality of the raw reads was assessed using FastQC (http://www.bioinformatics.babraham.ac.uk/projects/fastqc/). Sequencing adapters were trimmed from both pairs of raw reads using Cutadapt (Martin 2011). The filtered reads from EF-pool and LF-pool were aligned to the HAL2 genome v2.0 assembly using the bwa mem algorithm with default parameters (Li and Durbin 2009). SNP-calling was performed by the mpileup function from bcftools (Li 2011). A SNP-index, which is the proportion of SNPs that have different alleles other than the HAL2 reference alleles, was calculated for both EF and LF pool separately following the method described before (Takagi et al. 2013). ΔSNP-index, which is the difference of the proportion of alternative allele between two pools was calculated by subtracting the SNP-index of the EF pool from the SNP-index of the LF pool. The average distributions of the SNP-index and ΔSNP-index for a given genomic interval was estimated by using a sliding window approach with 1 Mb window size and 10 kb step. Confidence intervals were obtained by simulating a F_2_ mapping population with the same pool size of this bulk segregating population with 10,000 replications for a given sequence depth. This process was replicated for a range of sequence depths in order to obtain confidence interval (CI) precisely for a given sequence depth. The SNP-index graphs for EF-pool and LF-pool, as well as corresponding ΔSNP-index graph were plotted using ggplot2 package in R. Genomic intervals that crossed the 95% CI threshold of ΔSNP-index were considered as candidate genomic regions harboring a locus associated with flowering time.

### Phenotypic Evaluation

Broad-sense heritability (*H^2^*) was calculated as *V_g_*/*V_p_* using the Sommer package in R, where *V_g_* is estimated from the kinship matrix as genetic variance and *V_p_* is the total phenotypic variance (Covarrubias-Pazaran 2016). For genetic correlation estimation, flowering time from three seasonal experiments was used as response variables and similarly the kinship matrix was modeled as a random effect and used to estimate the additive genetic covariance. For G × E interactions on flowering time, we used a likelihood-ratio test to compare the following two models: 1) a main effect model assumed that there is no G × E and 2) unstructured model assumed that there are G × E interactions and unstructured variance-covariance matrix for the different environments. Significance of the likelihood-ratio test for G × E interactions was assessed at the level of α = 0.05.

### Tests of Selection Based on Segregation in a Cross

Fraser (2020) noted that the pattern of trait variance in recombinant populations can provide insight into whether the parental trait divergence is consistent with neutral divergence or more likely the result of natural selection. The approach is a generalization of the QTL sign test (Orr 1998) and rests on the realization that the phenotypic distribution in a segregating/recombinant population can be treated as a null model for the distribution of phenotypes expected under neutral evolution. In this framework, the pattern of underlying QTL effects under strong directional selection is expected to be complementary (in a consistent direction) if the trait has experienced consistent strong directional selection leading to parental divergence, whereas a mix of positive and negative effects is expected if the trait evolved by neutral processes. By extension, if a trait has experienced strong directional selection driving parental divergence the variance among parental lines is expected to be larger than the segregating variance in the recombinant population. The *v*-test was performed as described in equation 2 of Fraser (2020) for our genetic populations. In brief, to calculate *v,* we first estimated the trait variances within and between parents of the cross. Then, we estimated the variance among F_2_ and RIL means and used a *c* value of 2 for F_2_ and 1 for RIL (Fraser 2020).

### QTL Analysis in RIL Population

The RIL linkage map was built as previously described using the HAL2 genome assembly as the reference. Given our experiments were conducted in different environments, we completed QTL mapping analyses to test for QTL × environment interactions. We compared the likelihood of models allowing QTL to interact with the environment versus reduced models adjusting only for the additive effect of the environment using the R/qtl2 package (Broman et al. 2019). The full model is expressed as: phenotype = µ + QTL + E + QTL × E + kinship + e, and the reduced model is: phenotype = µ + QTL + E + kinship + e, where µ is the population mean, QTL is the marker effects, E is the environmental effects, QTL × E is the interaction between marker and environmental effects, and e is the error terms. The genome scan was accomplished through “scan1” function. The statistical significance of the genome scan was established by performing a stratified permutation test (*n* = 1,000) for the full model and reduced model using “scan1perm” function. The difference of thresholds between these two models from 1,000 permutations was considered as the threshold for the QTL × E model. The estimated QTL effect was obtained using “scan1coef” function.

QTL mapping for each RIL experiment was completed in R using a multiple-QTL model in the R/qtl package (Broman et al. 2003). The “scantwo” function with 1,000 permutations was used to calculate penalties for main effect and interactions for each flowering time trait. The “stepwiseqtl” function was used to conduct a forward-backward search with default setting of maximum number of QTL and account for epistasis that optimized the penalized LOD score criterion. Threshold values for type 1 error rates were set at α = 0.05 for flowering time traits were based on permutations. 1.5 LOD drop intervals of QTL were calculated using the “qtlStats” function.

### QTL Fine-Mapping

We identified a single RIL line (FH-312) that was heterozygous across the 2-LOD support interval of *qFT-5* but homozygous for either HAL2 or FIL2 alleles at the majority of the remainder of the genome. Therefore, this RIL line can be applied to validation of QTL through a HIF strategy (Tuinstra et al. 1997). We developed a HIF population containing 1,668 F_2_ progenies by self-fertilization of FH-312. Two flanking markers M5-7948 and M5-12422 were used to screen the HIF progenies and identify recombinants. Selfed progeny from selected recombinants were generated to a F_3_ population. Eight additional markers between markers M5-7948 and M5-12422 were used to genotype all recombinant-derived F_3_ individuals. Flowering time of individual plants from the recombinant-derived F_3_ population was scored. Individual marker effects on flowering time were tested using an ANOVA while controlling for initial seedling height. Finally, a pair of NILs (NIL^qFT-5-FIL2^ and NIL^qFT-5-HAL2^) were developed by selfing a heterozygous plant (HIF-2-2-1) and screening with markers to confirm homozygous FIL2 and HAL2 genotypes across the *qFT-5* interval. Primers used for all marker amplification are listed in supplementary table S3, Supplementary Material online.

### Reciprocal Transplant Field Experiments at Coastal and Inland Sites

We used a reciprocal transplant experiment to study QTL allelic effects near the location of origins of the HAL2 and FIL2 parental lines. Our reciprocal transplant experiment was initiated with seedlings. Seeds of HAL2 and FIL2 parental lines and the pair of NILs (NIL^qFT-5-FIL2^ and NIL^qFT-5-HAL2^) created from fine-mapping experiments were scarified by sandpaper and placed on moistened sand in Petri dishes with a 12 h-light and 12 h-dark photoperiod at 25°C in the growth chamber. Five days after germination, 50 individual seedlings of each genotype were maintained with a randomized design on the bench of the greenhouse at the Brackenridge Field Laboratory at the University of Texas at Austin. Ten days after germination, all seedlings were transplanted to randomized positions in a rectangular array with individual plants separated by 1 m in the field at the UT Brackenridge Field Laboratory in Austin, TX and Kika de la Garza USDA Plant Materials Center in Kingsville, TX on April 5, 2019 and April 7, 2019. We measured a number of traits related to growth and development including tiller number, primary branch number, secondary branch number, number of flowers per panicle, and above-ground biomass at the end of the growing season and associated with fall senescence. We estimated the total growing season fitness from multiplying tillers × number of flowers per panicle. We used a two-way ANOVA to examine the effect of *qFT-5* and site on fitness-related traits and total fitness recorded in the field. To improve normality of residuals and homogeneity of variances, total fitness was square-root-transformed before analysis. When the QTL × site interaction was statistically significant, contrasts were used to examine differences between the local and nonlocal *qFT-5* alleles separately by site.

### DNA Extraction and Molecular Marker Analysis

Genomic DNA was isolated using the modified CTAB method from young leaves of F_2_ plants and HIF plants for QTL analysis (Murray and Thompson 1980). InDel markers were developed based on *P. hallii var.* HAL2 genome v2.0 and *P. hallii var.* FIL2 genome v3.0 assembly (https://phytozome.jgi.doe.gov/pz/portal.html). Primers were designed using software Primer 3.0 (http://bioinfo.ut.ee/primer3-0.4.0/) and were then amplified from F_2_ individuals and HIF individuals. A total volume of 10 µl reaction mixture was used for PCR amplification, which is composed 1 µl of template DNA, 0.3 µl of each primer (10 mM), 2 µl 5 × Buffer, 1.2 µl MgCl_2_, 1 µl dNTP, and 0.1 µl GoTag (Promega). Amplification was performed on program for the initial denaturing step with 96°C for 3 min, followed by 38 cycles for 30 s at 96°C, 30 s at 58°C, 30 s at 72°C, with a final extension at 72°C for 5 min. The PCR products can be well separated using 2.5% agarose gel electrophoresis.

### RNA Extraction, Transcriptome and Quantitative RT-PCR Analysis

The expression level of *PhFTL9* in leaves was measured by transcriptome data and quantitative RT-PCR (qPCR). For transcriptome analysis, leaf tissues were harvested in the field condition at the J.J. Pickle Research Campus at the University of Texas at Austin. We collected 13 FIL2 replicates and 12 HAL2 replicates at dusk and stored these samples immediately in liquid nitrogen. Libraries were prepared using a TagSeq protocol as described before (Weng et al. 2019). Prepared libraries were then submitted to the Genomic Sequencing and Analysis Facility at the University of Texas at Austin to obtain 3–5 million reads per sample (SE 100 BP) on Illumina HiSeq 2500 platform. Bioinformatic analysis of TagSeq data was performed as described before (Weng et al. 2019). The filtered reads were aligned to the *P. hallii* HAL2 v2.1 reference (https://phytozome-next.jgi.doe.gov/info/PhalliiHAL_v2_1) using BWA-mem with default parameters. Variance stabilized transformed counts were used to compare the expression level of genes in the 380-kb qFT-5 interval between HAL2 and FIL2 (supplementary table S6, Supplementary Material online). For qPCR analysis, HAL2 and FIL2 individuals were established with a 14 h-light and 10 h-dark photoperiod at 25°C under greenhouse conditions. Leaf samples were harvested 2 weeks post germination at 08:00 (zeitgeber time 2) and 18:00 (zeitgeber time 12). We collected the samples from four different plants as three biological replicates for each genotype and stored these samples in liquid nitrogen. For qPCR, we isolated total RNA from the leaves using an RNA extraction kit (TRIzol reagent, Invitrogen). 2 µg total RNA was reverse-transcribed using SSII reverse transcriptase (Invitrogen) in a volume of 80 µl to obtain cDNA. We used primers qPhFTL9-F and qPhFTL9-R for amplifying the transcript of *PhFTL9*. We used primers qPhUBI-F and qPhUBI-R for ubiquitin-conjugating enzyme (Pahal.3G250900) as the internal control (Lovell et al. 2018). We carried out qPCR in a total volume of 10 μl containing 2 μl of the reverse-transcribed product above, 0.25 μM gene-specific primers and 5 μl LightCycler 480 SYBR Green I Master (Roche) on a Roche LightCycler 480 II real-time PCR System according to the manufacturer’s instructions. The relative expression levels of *PhFTL9* were obtained using the delta-delta Ct method (Livak and Schmittgen 2001). Primers used for qPCR analysis are listed in supplementary table S3, Supplementary Material online.

### Sequence Analysis

To evaluate protein evolution, we identified the protein-coding sequences of *PhFTL9* orthologues in *P. hallii* HAL2 (PhHAL.5G159600), *P. hallii* FIL2 (Pahal.5G160000), *P. virgatum* (Pavir.5KG539100), *Setaria italica* (Seita.5G317600), *Z. mays* (Zm00001d043461_T001), and *Sorghum bicolor* (Sobic.003G295300) from Phytozome v13 (https://phytozome-next.jgi.doe.gov/). We calculated *K_a_*, *K_s_*, and *K_a_*/*K_s_* values using the software K_a_K_s__Calculator 2.0 with a modified version of the Yang and Nielsen method (Wang et al. 2010). To explore the promoter evolution, the EARS (Evolutionary Analysis of Regulatory Sequences), a robust and highly sensitive alignment-based approach, was employed to search for evolutionarily conserved regions in the putative promoter regions of *PhFTL9* and its homologs in *P. virgatum*, *Setaria italica* (Picot et al. 2010). The promoter region from the HAL2 genome v2.0 assembly was selected as the reference for EARS analysis. We used a total of 59 accessions from *P. hallii* population (49 inland accessions and 10 coastal accessions) (Lovell et al. 2018) for promoter divergence analysis. In order to obtain high quality alignment, we chose to map each genetic cluster separately to their closest reference genome therefore inland accessions were mapped to inland reference genome (*P. hallii var.* HAL2 genome v2.0) and coastal accessions were mapped to coastal reference genome (*P. hallii var.* FIL2 genome v3.0). Variant calling was carried out separately for each genetic cluster using the Genome Analysis Toolkit (GATK) version 3.8.1 and SNP and InDel variants were filtered separately based on the hard filtering recommendation by GATK. Codes for the pipeline can be found in GitHub (https://github.com/tahia/SNP_calling_GATK). To compare *FTL9* promoter divergence statistics (Tajima’s D and Pi) between inland and coastal genetic cluster against the random genomic background, we obtained a set of random 1,000 promoter pair from one-to-one orthologues of inland and coastal populations. We defined upstream 1,000 bp promoter sequence from the TSS of a gene as the core promoter region and upstream 1,001–2,000 bp promoter sequence as the proximal promoter region. For a given genetic cluster, the divergence statistics were estimated for these core and proximal promoter regions separately. Tajima’s D was calculated by VCFtools (Danecek et al. 2011) for variant sites of a given gene and Pi was estimated by pixy (Korunes and Samuk 2021) for all detectable sites for a target gene.

## Supplementary Material

msac203_Supplementary_Data

## Data Availability

Raw reads for the QTL-seq experiment are available in the Sequence Read Archive database (https://www.ncbi.nlm.nih.gov/sra) with accession numbers SRR14049075 and SRR14049076.

## References

[msac203-B1] Agren J , Oakley CG, Lundemo S, Schemske DW. 2017. Adaptive divergence in flowering time among natural populations of *Arabidopsis thaliana*: estimates of selection and QTL mapping. Evolution. 71:550–564. 27859214 10.1111/evo.13126

[msac203-B2] Agren J , Oakley CG, McKay JK, Lovell JT, Schemske DW. 2013. Genetic mapping of adaptation reveals fitness tradeoffs in *Arabidopsis thaliana*. Proc Natl Acad Sci USA. 110:21077–21082. 24324156 10.1073/pnas.1316773110 PMC3876199

[msac203-B3] Anderson JT , Lee CR, Mitchell-Olds T. 2011. Life-history QTLs and natural selection on flowering time in *Boechera Stricta*, a perennial relative of *Arabidopsis*. Evolution. 65:771–787. 21083662 10.1111/j.1558-5646.2010.01175.x PMC3155413

[msac203-B4] Anderson JT , Lee CR, Rushworth CA, Colautti RI, Mitchell-Olds T. 2013. Genetic trade-offs and conditional neutrality contribute to local adaptation. Mol Ecol. 22:699–708. 22420446 10.1111/j.1365-294X.2012.05522.x PMC3492549

[msac203-B5] Anderson JT , Willis JH, Mitchell-Olds T. 2011. Evolutionary genetics of plant adaptation. Trends Genet. 27:258–266. 21550682 10.1016/j.tig.2011.04.001 PMC3123387

[msac203-B6] Auge GA , Penfield S, Donohue K. 2019. Pleiotropy in developmental regulation by flowering-pathway genes: is it an evolutionary constraint? New Phytol. 224:55–70. 31074008 10.1111/nph.15901

[msac203-B7] Blackman BK . 2017. Changing responses to changing seasons: natural variation in the plasticity of flowering time. Plant Physiol. 173:16–26. 27872243 10.1104/pp.16.01683 PMC5210766

[msac203-B8] Blackman BK , Strasburg JL, Raduski AR, Michaels SD, Rieseberg LH. 2010. The role of recently derived *FT* paralogs in sunflower domestication. Curr Biol. 20:629–635. 20303265 10.1016/j.cub.2010.01.059 PMC2898918

[msac203-B9] Bouche F , Lobet G, Tocquin P, Perilleux C. 2016. FLOR-ID: an interactive database of flowering-time gene networks in *Arabidopsis thaliana*. Nucleic Acids Res. 44:D1167–D1171. 26476447 10.1093/nar/gkv1054 PMC4702789

[msac203-B10] Broman KW , Gatti DM, Simecek P, Furlotte NA, Prins P, Sen S, Yandell BS, Churchill GA. 2019. R/qtl2: software for mapping quantitative trait loci with high-dimensional data and multiparent populations. Genetics. 211:495–502. 30591514 10.1534/genetics.118.301595 PMC6366910

[msac203-B11] Broman KW , Wu H, Sen S, Churchill GA. 2003. R/qtl: QTL mapping in experimental crosses. Bioinformatics. 19:889–890. 12724300 10.1093/bioinformatics/btg112

[msac203-B12] Chiang GCK , Barua D, Kramer EM, Amasino RM, Donohue K. 2009. Major flowering time gene, *FLOWERING LOCUS C*, regulates seed germination in *Arabidopsis thaliana*. Proc Natl Acad Sci USA. 106:11661–11666. 19564609 10.1073/pnas.0901367106 PMC2710639

[msac203-B13] Covarrubias-Pazaran G . 2016. Genome-assisted prediction of quantitative traits using the R package *sommer*. PLoS ONE. 11:e0156744. 10.1371/journal.pone.0156744 PMC4894563 27271781

[msac203-B14] Danecek P , Auton A, Abecasis G, Albers CA, Banks E, DePristo MA, Handsaker RE, Lunter G, Marth GT, Sherry ST, et al 2011. The variant call format and VCFtools. Bioinformatics 27:2156–2158. 21653522 10.1093/bioinformatics/btr330 PMC3137218

[msac203-B15] Doi K , Izawa T, Fuse T, Yamanouchi U, Kubo T, Shimatani Z, Yano M, Yoshimura A. 2004. *Ehd1*, a B-type response regulator in rice, confers short-day promotion of flowering and controls *FT-Iike* gene expression independently of *Hd1*. Genes Dev. 18:926–936. 15078816 10.1101/gad.1189604 PMC395851

[msac203-B16] Elzinga JA , Atlan A, Biere A, Gigord L, Weis AE, Bernasconi G. 2007. Time after time: flowering phenology and biotic interactions. Trends Ecol Evol. 22:432–439. 17573151 10.1016/j.tree.2007.05.006

[msac203-B17] Fishman L , Sweigart AL, Kenney AM, Campbell S. 2014. Major quantitative trait loci control divergence in critical photoperiod for flowering between selfing and outcrossing species of monkeyflower (*Mimulus*). New Phytol. 201:1498–1507. 24304557 10.1111/nph.12618

[msac203-B18] Forsythe WC , Rykiel EJ, Stahl RS, Wu HI, Schoolfield RM. 1995. A model comparison for daylength as a function of latitude and day of year. Ecol Modell. 80:87–95.

[msac203-B19] Fournier-Level A , Korte A, Cooper MD, Nordborg M, Schmitt J, Wilczek AM. 2011. A map of local adaptation in *Arabidopsis thaliana*. Science. 334:86–89. 21980109 10.1126/science.1209271

[msac203-B20] Fraser HB . 2020. Detecting selection with a genetic cross. Proc Natl Acad Sci USA. 117:22323–22330. 32848059 10.1073/pnas.2014277117 PMC7486746

[msac203-B21] Fry JD . 1996. The evolution of host specialization: are trade-offs overrated? Am Nat. 148:S84–S107.

[msac203-B22] Galbiati F , Chiozzotto R, Locatelli F, Spada A, Genga A, Fornara F. 2016. *Hd3a*, *RFT1* and *Ehd1* integrate photoperiodic and drought stress signals to delay the floral transition in rice. Plant Cell Environ. 39:1982–1993. 27111837 10.1111/pce.12760

[msac203-B23] Grabowski PP , Evans J, Daum C, Deshpande S, Barry KW, Kennedy M, Ramstein G, Kaeppler SM, Buell CR, Jiang Y, et al 2017. Genome-wide associations with flowering time in switchgrass using exome-capture sequencing data. New Phytol. 213:154–169. 27443672 10.1111/nph.14101

[msac203-B24] Guo L , Wang X, Zhao M, Huang C, Li C, Li D, Yang CJ, York AM, Xue W, Xu G, et al 2018. Stepwise cis-regulatory changes in *ZCN8* contribute to maize flowering-time adaptation. Curr Biol. 28:3005–3015.e4. 30220503 10.1016/j.cub.2018.07.029 PMC6537595

[msac203-B25] Hall MC , Lowry DB, Willis JH. 2010. Is local adaptation in *Mimulus guttatus* caused by trade-offs at individual loci? Mol Ecol. 19:2739–2753. 20546131 10.1111/j.1365-294X.2010.04680.x PMC11104436

[msac203-B26] Hall MC , Willis JH. 2006. Divergent selection on flowering time contributes to local adaptation in *Mimulus guttatus* populations. Evolution. 60:2466–2477. 17263109

[msac203-B27] Hayama R , Coupland G. 2004. The molecular basis of diversity in the photoperiodic flowering responses of *Arabidopsis* and rice. Plant Physiol. 135:677–684. 15208414 10.1104/pp.104.042614 PMC514104

[msac203-B28] Hiraoka K , Yamaguchi A, Abe M, Araki T. 2013. The florigen genes *FT* and *TSF* modulate lateral shoot outgrowth in *Arabidopsis thaliana*. Plant Cell Physiol. 54:352–368. 23220822 10.1093/pcp/pcs168

[msac203-B29] Ho WW , Weigel D. 2014. Structural features determining flower-promoting activity of *Arabidopsis FLOWERING LOCUS T*. Plant Cell. 26:552–564. 24532592 10.1105/tpc.113.115220 PMC3967025

[msac203-B30] Izawa T . 2007. Adaptation of flowering-time by natural and artificial selection in *Arabidopsis* and rice. J Exp Bot. 58:3091–3097. 17693414 10.1093/jxb/erm159

[msac203-B31] Kawecki TJ , Ebert D. 2004. Conceptual issues in local adaptation. Ecol Lett. 7:1225–1241.

[msac203-B32] Khasanova A , Lovell JT, Bonnette J, Weng X, Jenkins J, Yoshinaga Y, Schmutz J, Juenger TE. 2019. The genetic architecture of shoot and root trait divergence between mesic and xeric ecotypes of a perennial grass. Front Plant Sci. 10:366. 31019518 10.3389/fpls.2019.00366 PMC6458277

[msac203-B33] Kim DH , Doyle MR, Sung S, Amasino RM. 2009. Vernalization: winter and the timing of flowering in plants. Annu Rev Cell Dev Biol. 25:277–299. 19575660 10.1146/annurev.cellbio.042308.113411

[msac203-B34] Kojima S , Takahashi Y, Kobayashi Y, Monna L, Sasaki T, Araki T, Yano M. 2002. *Hd3a*, a rice ortholog of the *Arabidopsis FT* gene, promotes transition to flowering downstream of *Hd1* under short-day conditions. Plant Cell Physiol. 43:1096–1105. 12407188 10.1093/pcp/pcf156

[msac203-B35] Komiya R , Ikegami A, Tamaki S, Yokoi S, Shimamoto K. 2008. *Hd3a* and *RFT1* are essential for flowering in rice. Development. 135:767–774. 18223202 10.1242/dev.008631

[msac203-B36] Koo BH , Yoo SC, Park JW, Kwon CT, Lee BD, An G, Zhang Z, Li J, Li Z, Paek NC. 2013. Natural variation in *OsPRR37* regulates heading date and contributes to rice cultivation at a wide range of latitudes. Mol Plant. 6:1877–1888. 23713079 10.1093/mp/sst088

[msac203-B37] Korunes KL , Samuk K. 2021. pixy: Unbiased estimation of nucleotide diversity and divergence in the presence of missing data. Mol Ecol Resour. 21:1359–1368. 33453139 10.1111/1755-0998.13326 PMC8044049

[msac203-B38] Larios E , Venable DL. 2018. Selection for seed size: the unexpected effects of water availability and density. Funct Ecol. 32:2216–2224.

[msac203-B39] Leimu R , Fischer M. 2008. A meta-analysis of local adaptation in plants. PLoS ONE. 3:e4010. 19104660 10.1371/journal.pone.0004010 PMC2602971

[msac203-B40] Li H . 2011. A statistical framework for SNP calling, mutation discovery, association mapping and population genetical parameter estimation from sequencing data. Bioinformatics 27:2987–2993. 21903627 10.1093/bioinformatics/btr509 PMC3198575

[msac203-B41] Li H , Durbin R. 2009. Fast and accurate short read alignment with Burrows-Wheeler transform. Bioinformatics 25:1754–1760. 19451168 10.1093/bioinformatics/btp324 PMC2705234

[msac203-B42] Liu L , Adrian J, Pankin A, Hu J, Dong X, von Korff M, Turck F. 2014. Induced and natural variation of promoter length modulates the photoperiodic response of *FLOWERING LOCUS T*. Nat Commun. 5:4558. 25087553 10.1038/ncomms5558 PMC4143923

[msac203-B43] Livak KJ , Schmittgen TD. 2001. Analysis of relative gene expression data using real-time quantitative PCR and the 2(-Delta Delta C(T)) method. Methods. 25:402–408. 11846609 10.1006/meth.2001.1262

[msac203-B44] Lovell JT , Jenkins J, Lowry DB, Mamidi S, Sreedasyam A, Weng XY, Barry K, Bonnette J, Campitelli B, Daum C, et al 2018. The genomic landscape of molecular responses to natural drought stress in *Panicum hallii*. Nat Commun. 9:5213. 30523281 10.1038/s41467-018-07669-x PMC6283873

[msac203-B45] Lovell JT , Juenger TE, Michaels SD, Lasky JR, Platt A, Richards JH, Yu XH, Easlon HM, Sen S, Mckay JK. 2013. Pleiotropy of *FRIGIDA* enhances the potential for multivariate adaptation. Proc Royal Soc B Biol Sci. 280:20131043. 10.1098/rspb.2013.1043 PMC3774242 23698015

[msac203-B46] Lovell JT , Schwartz S, Lowry DB, Shakirov EV, Bonnette JE, Weng XY, Wang M, Johnson J, Sreedasyam A, Plott C, et al 2016. Drought responsive gene expression regulatory divergence between upland and lowland ecotypes of a perennial C_4_ grass. Genome Res. 26:510–518. 26953271 10.1101/gr.198135.115 PMC4817774

[msac203-B47] Lowry DB , Hernandez K, Taylor SH, Meyer E, Logan TL, Barry KW, Chapman JA, Rokhsar DS, Schmutz J, Juenger TE. 2015. The genetics of divergence and reproductive isolation between ecotypes of *Panicum hallii*. New Phytol. 205:402–414. 25252269 10.1111/nph.13027 PMC4265272

[msac203-B48] Lowry DB , Logan TL, Santuari L, Hardtke CS, Richards JH, DeRose-Wilson LJ, McKay JK, Sen S, Juenger TE. 2013. Expression quantitative trait locus mapping across water availability environments reveals contrasting associations with genomic features in *Arabidopsis*. Plant Cell. 25:3266–3279. 24045022 10.1105/tpc.113.115352 PMC3809531

[msac203-B49] Lowry DB , Lovell JT, Zhang L, Bonnette J, Fay PA, Mitchell RB, Lloyd-Reilley J, Boe AR, Wu YQ, Rouquette FM, et al 2019. QTL × environment interactions underlie adaptive divergence in switchgrass across a large latitudinal gradient. Proc Natl Acad Sci USA. 116:12933–12941. 31182579 10.1073/pnas.1821543116 PMC6600931

[msac203-B50] Lowry DB , Purmal CT, Meyer E, Juenger TE. 2012. Microsatellite markers for the native Texas perennial grass, *Panicum hallii* (Poaceae). Am J Bot. 99:e114–e116. 22362543 10.3732/ajb.1100430

[msac203-B51] Martin M . 2011. Cutadapt removes adapter sequences from high-throughput sequencing reads. EMBnet J. 17:10–12.

[msac203-B52] Mee JA , Yeaman S. 2019. Unpacking conditional neutrality: genomic signatures of selection on conditionally beneficial and conditionally deleterious mutations. Am Nat. 194:529–540. 31490722 10.1086/702314

[msac203-B53] Meng X , Muszynski MG, Danilevskaya ON. 2011. The *FT-Like ZCN8* gene functions as a floral activator and is involved in photoperiod sensitivity in maize. Plant Cell. 23:942–960. 21441432 10.1105/tpc.110.081406 PMC3082274

[msac203-B54] Mitchell-Olds T , Willis JH, Goldstein DB. 2007. Which evolutionary processes influence natural genetic variation for phenotypic traits? Nat Rev Genet. 8:845–856. 17943192 10.1038/nrg2207

[msac203-B55] Murphy RL , Klein RR, Morishige DT, Brady JA, Rooney WL, Miller FR, Dugas DV, Klein PE, Mullet JE. 2011. Coincident light and clock regulation of *pseudoresponse regulator protein 37* (*PRR37*) controls photoperiodic flowering in sorghum. Proc Natl Acad Sci U S A. 108:16469–16474. 21930910 10.1073/pnas.1106212108 PMC3182727

[msac203-B56] Murray MG , Thompson WF. 1980. Rapid isolation of high molecular weight plant DNA. Nucleic Acids Res. 8:4321–4325. 7433111 10.1093/nar/8.19.4321 PMC324241

[msac203-B57] Niwa M , Daimon Y, Kurotani K, Higo A, Pruneda-Paz JL, Breton G, Mitsuda N, Kay SA, Ohme-Takagi M, Endo M, et al 2013. BRANCHED1 interacts with FLOWERING LOCUS T to repress the floral transition of the axillary meristems in *Arabidopsis*. Plant Cell. 25:1228–1242. 23613197 10.1105/tpc.112.109090 PMC3663264

[msac203-B58] Orr HA . 1998. Testing natural selection *vs.* genetic drift in phenotypic evolution using quantitative trait locus data. Genetics. 149:2099–2104. 9691061 10.1093/genetics/149.4.2099 PMC1460271

[msac203-B59] Palacio-Mejia JD , Grabowski PP, Ortiz EM, Silva-Arias GA, Haque T, Des Marais DL, Bonnette J, Lowry DB, Juenger TE. 2021. Geographic patterns of genomic diversity and structure in the C_4_ grass *Panicum hallii* across its natural distribution. AoB Plants. 13:plab002. 10.1093/aobpla/plab002 PMC7937184 33708370

[msac203-B60] Picot E , Krusche P, Tiskin A, Carre I, Ott S. 2010. Evolutionary analysis of regulatory sequences (EARS) in plants. Plant J. 64:165–176. 20659275 10.1111/j.1365-313X.2010.04314.x

[msac203-B61] Primack RB , Kang H. 1989. Measuring fitness and natural selection in wild plant populations. Annu Rev Ecol Evol Syst. 20:367–396.

[msac203-B62] Putterill J , Robson F, Lee K, Simon R, Coupland G. 1995. The *CONSTANS* gene of *Arabidopsis* promotes flowering and encodes a protein showing similarities to zinc finger transcription factors. Cell. 80:847–857. 7697715 10.1016/0092-8674(95)90288-0

[msac203-B63] Qin Z , Bai Y, Muhammad S, Wu X, Deng P, Wu J, An H, Wu L. 2019. Divergent roles of FT-like 9 in flowering transition under different day lengths in *Brachypodium distachyon*. Nat Commun. 10:812. 30778068 10.1038/s41467-019-08785-y PMC6379408

[msac203-B64] Razzaque S , Juenger TE. 2021. The ecology and quantitative genetics of seed and seedling traits in upland and lowland ecotypes of a perennial grass. bioRxiv. 2021.12.14.472686. 10.1002/evl3.297 PMC9783394 36579162

[msac203-B65] Salome PA , Bomblies K, Laitinen RA, Yant L, Mott R, Weigel D. 2011. Genetic architecture of flowering-time variation in *Arabidopsis thaliana*. Genetics. 188:421–433. 21406681 10.1534/genetics.111.126607 PMC3122318

[msac203-B66] Sandring S , Agren J. 2009. Pollinator-mediated selection on floral display and flowering time in the perennial herb *Arabidopsis Lyrata*. Evolution. 63:1292–1300. 19154392 10.1111/j.1558-5646.2009.00624.x

[msac203-B67] Savolainen O , Lascoux M, Merila J. 2013. Ecological genomics of local adaptation. Nat Rev Genet. 14:807–820. 24136507 10.1038/nrg3522

[msac203-B68] Scarcelli N , Cheverud JM, Schaal BA, Kover PX. 2007. Antagonistic pleiotropic effects reduce the potential adaptive value of the *FRIGIDA* locus. Proc Natl Acad Sci U S A. 104:16986–16991. 17940010 10.1073/pnas.0708209104 PMC2040464

[msac203-B69] Schwartz C , Balasubramanian S, Warthmann N, Michael TP, Lempe J, Sureshkumar S, Kobayashi Y, Maloof JN, Borevitz JO, Chory J, et al 2009. *Cis*-regulatory changes at *FLOWERING LOCUS T* mediate natural variation in flowering responses of *Arabidopsis thaliana*. Genetics. 183:723–732. 19652183 10.1534/genetics.109.104984 PMC2766330

[msac203-B70] Takagi H , Abe A, Yoshida K, Kosugi S, Natsume S, Mitsuoka C, Uemura A, Utsushi H, Tamiru M, Takuno S, et al 2013. QTL-seq: rapid mapping of quantitative trait loci in rice by whole genome resequencing of DNA from two bulked populations. Plant J. 74:174–183. 23289725 10.1111/tpj.12105

[msac203-B71] Tamaki S , Matsuo S, Wong HL, Yokoi S, Shimamoto K. 2007. *Hd3a* protein is a mobile flowering signal in rice. Science. 316:1033–1036. 17446351 10.1126/science.1141753

[msac203-B72] Taylor MA , Wilczek AM, Roe JL, Welch SM, Runcie DE, Cooper MD, Schmitt J. 2019. Large-effect flowering time mutations reveal conditionally adaptive paths through fitness landscapes in *Arabidopsis thaliana*. Proc Natl Acad Sci U S A. 116:17890–17899. 31420516 10.1073/pnas.1902731116 PMC6731683

[msac203-B73] Tiffin P , Ross-Ibarra J. 2014. Advances and limits of using population genetics to understand local adaptation. Trends Ecol Evol. 29:673–680. 25454508 10.1016/j.tree.2014.10.004

[msac203-B74] Tiwari SB , Shen Y, Chang HC, Hou YL, Harris A, Ma SF, McPartland M, Hymus GJ, Adam L, Marion C, et al 2010. The flowering time regulator CONSTANS is recruited to the *FLOWERING LOCUS T* promoter via a unique *cis*-element. New Phytol. 187:57–66. 20406410 10.1111/j.1469-8137.2010.03251.x

[msac203-B75] Tsuji H , Tachibana C, Tamaki S, Taoka K, Kyozuka J, Shimamoto K. 2015. Hd3a promotes lateral branching in rice. Plant J. 82:256–266. 25740115 10.1111/tpj.12811

[msac203-B76] Tuinstra MR , Ejeta G, Goldsbrough PB. 1997. Heterogeneous inbred family (HIF) analysis: a method for developing near-isogenic lines that differ at quantitative trait loci. Theor Appl Genet. 95:1005–1011.

[msac203-B77] Turck F , Fornara F, Coupland G. 2008. Regulation and identity of florigen: FLOWERING LOCUS T moves center stage. Annu Rev Plant Biol. 59:573–594. 18444908 10.1146/annurev.arplant.59.032607.092755

[msac203-B78] Turner AS , Faure S, Zhang Y, Laurie DA. 2013. The effect of day-neutral mutations in barley and wheat on the interaction between photoperiod and vernalization. Theor Appl Genet. 126(9):2267–2277. 23737074 10.1007/s00122-013-2133-6 PMC3755224

[msac203-B79] Verhoeven KJF , Poorter H, Nevo E, Biere A. 2008. Habitat-specific natural selection at a flowering-time QTL is a main driver of local adaptation in two wild barley populations. Mol Ecol. 17:3416–3424. 18573164 10.1111/j.1365-294X.2008.03847.x

[msac203-B80] Wadgymar SM , Daws SC, Anderson JT. 2017. Integrating viability and fecundity selection to illuminate the adaptive nature of genetic clines. Evol Lett. 1:26–39. 30283636 10.1002/evl3.3 PMC6121800

[msac203-B81] Wang J , Ding J, Tan B, Robinson KM, Michelson IH, Johansson A, Nystedt B, Scofield DG, Nilsson O, Jansson S, et al 2018. A major locus controls local adaptation and adaptive life history variation in a perennial plant. Genome Biol. 19:72. 29866176 10.1186/s13059-018-1444-y PMC5985590

[msac203-B82] Wang DP , Zhang YB, Zhang Z, Zhu J, Yu J. 2010. KaKs_Calculator 2.0: a toolkit incorporating gamma-series methods and sliding window strategies. Genomics Proteomics Bioinformatics. 8:77–80. 20451164 10.1016/S1672-0229(10)60008-3 PMC5054116

[msac203-B83] Weng XY , Lovell JT, Schwartz SL, Cheng CD, Haque T, Zhang L, Razzaque S, Juenger TE. 2019. Complex interactions between day length and diurnal patterns of gene expression drive photoperiodic responses in a perennial C_4_ grass. Plant Cell Environ. 42:2165–2182. 30847928 10.1111/pce.13546

[msac203-B84] Weng XY , Wang L, Wang J, Hu Y, Du H, Xu CG, Xing YZ, Li XH, Xiao JH, Zhang QF. 2014. *Grain number, plant height, and heading date7* is a central regulator of growth, development, and stress response. Plant Physiol. 164:735–747. 24390391 10.1104/pp.113.231308 PMC3912102

[msac203-B85] Wolabu TW , Zhang F, Niu L, Kalve S, Bhatnagar-Mathur P, Muszynski MG, Tadege M. 2016. Three *FLOWERING LOCUS T-like* genes function as potential florigens and mediate photoperiod response in sorghum. New Phytol. 210:946–959. 26765652 10.1111/nph.13834

[msac203-B86] Woods DP , Bednarek R, Bouche F, Gordon SP, Vogel JP, Garvin DF, Amasino RM. 2017. Genetic architecture of flowering-time variation in *Brachypodium distachyon*. Plant Physiol. 173:269–279. 27742753 10.1104/pp.16.01178 PMC5210718

[msac203-B87] Woods DP , Dong YX, Bouche F, Bednarek R, Rowe M, Ream T, Amasino RM. 2019. A florigen paralog is required for short-day vernalization in a pooid grass. eLife. 8:e42153. 30618375 10.7554/eLife.42153 PMC6324881

[msac203-B88] Xue WY , Xing YZ, Weng XY, Zhao Y, Tang WJ, Wang L, Zhou HJ, Yu SB, Xu CG, Li XH, et al 2008. Natural variation in *Ghd7* is an important regulator of heading date and yield potential in rice. Nat Genet. 40:761–767. 18454147 10.1038/ng.143

[msac203-B89] Yan WH , Liu HY, Zhou XC, Li QP, Zhang J, Lu L, Liu TM, Liu HJ, Zhang CJ, Zhang ZY, et al 2013. Natural variation in *Ghd7.1* plays an important role in grain yield and adaptation in rice. Cell Res. 23:969–971. 23507971 10.1038/cr.2013.43 PMC3698629

[msac203-B90] Yano M , Katayose Y, Ashikari M, Yamanouchi U, Monna L, Fuse T, Baba T, Yamamoto K, Umehara Y, Nagamura Y, et al 2000. *Hd1*, a major photoperiod sensitivity quantitative trait locus in rice, is closely related to the arabidopsis flowering time gene *CONSTANS*. Plant Cell. 12:2473–2483. 11148291 10.1105/tpc.12.12.2473 PMC102231

[msac203-B91] Yeaman S . 2022. Evolution of polygenic traits under global vs local adaptation. Genetics. 220:iyab134. 35134196 10.1093/genetics/iyab134 PMC8733419

[msac203-B92] Zhang ZH , Wang K, Guo L, Zhu YJ, Fan YY, Cheng SH, Zhuang JY. 2012. Pleiotropism of the photoperiod-insensitive allele of *Hd1* on heading date, plant height and yield traits in rice. PLoS ONE. 7:e52538. 23285081 10.1371/journal.pone.0052538 PMC3527549

[msac203-B93] Zhu YJ , Fan YY, Wang K, Huang DR, Liu WZ, Ying JZ, Zhuang JY. 2017. *Rice Flowering Locus T 1* plays an important role in heading date influencing yield traits in rice. Sci Rep. 7:4918. 28687802 10.1038/s41598-017-05302-3 PMC5501849

