# Supplementary material for: A Pleiotropic Flowering Time QTL Exhibits Gene-by-Environment Interaction for Fitness in a Perennial Grass
Source: Mol Biol Evol. 2022 Sep 23;39(10):msac203. doi: 10.1093/molbev/msac203 (PMC9550986; doi:10.1093/molbev/msac203)
Supplement: msac203_Supplementary_Data [file msac203_supplementary_data.zip › Weng_et_al_Supplementary_Files.pdf]

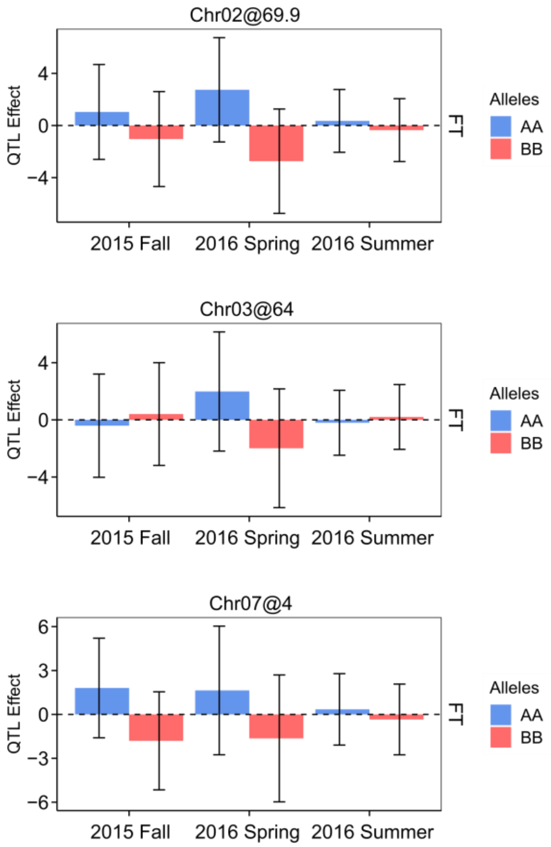

Supplementary Fig. S1. Genotypic effects of the five QTL with QTL × E detected in the full model. AA and BB indicate FIL2 and HAL2 alleles, respectively. These allelic effects are plotted side-by-side for each seasonal experiment. Positive additive effects indicate a delayed flowering time from that allele, while negative effects indicate an accelerated flowering time from that allele.

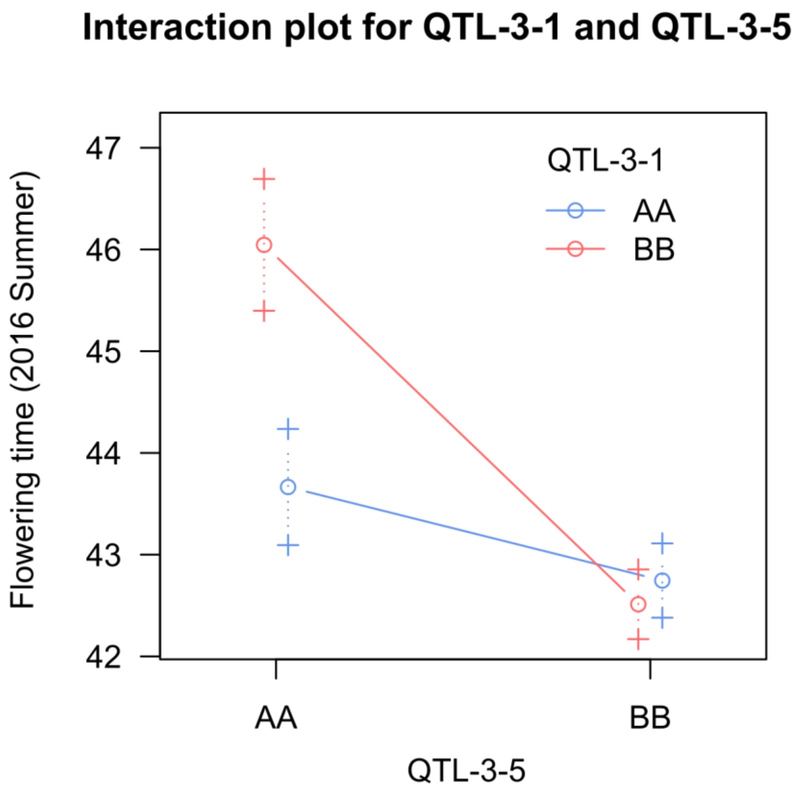

Supplementary Figure S2. Pairwise epistatic QTL in the 2016 summer RIL population. Plotted points indicate two-locus genotype means ± SE for the two loci containing flowering time between QTL-3-5 and QTL-3-1. AA and BB indicate homozygous FIL2 and HAL2 genotypes at relative QTL loci, respectively.

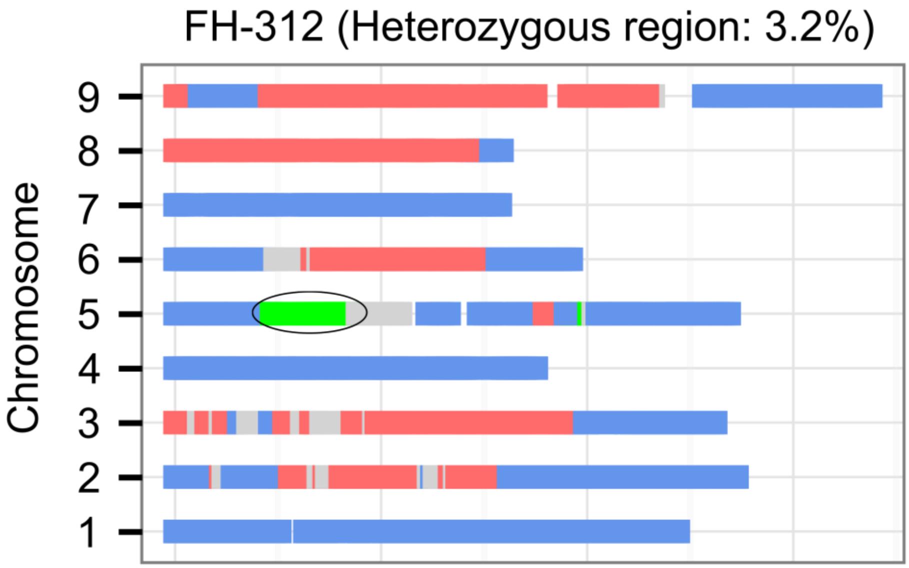

Supplementary Figure S3. Graphical genotypes of a heterogeneous inbred family (HIF) (FH-312) used for used for constructing Near Isogenic Lines(NILs) for fine mapping of *qFT-5*. Blue indicates regions homozygous for FIL2; red indicates regions homozygous for HAL2; green indicates heterozygous regions; grey indicates unknown regions. The *qFT-5* region are indicated by black circle.

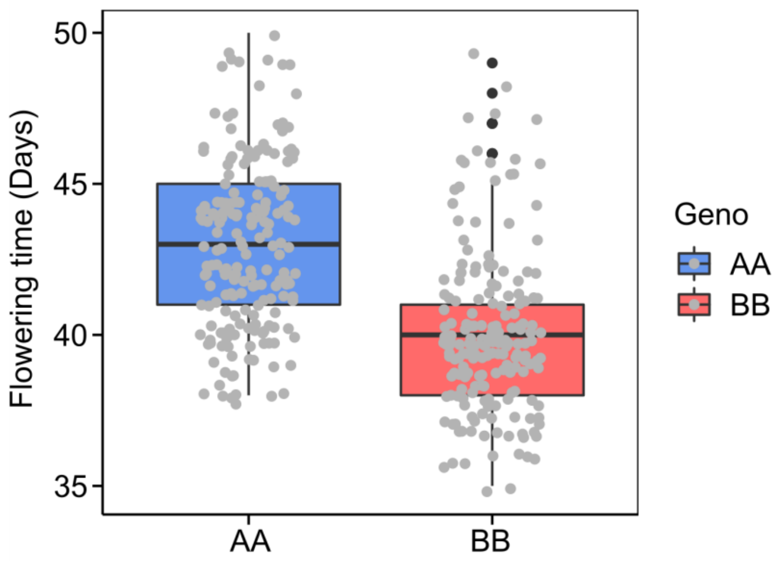

Supplementary Figure S4. The flowering time comparison between HIF progenies carrying FIL2 homozygous alleles at *qFT-5* region than those carrying HAL2 homozygous alleles. AA indicates the HIF progenies carrying homozygous FIL2 genotypes at two flanking markers (M5-7948 and M5-12422), while BB indicates the HIF progenies carrying homozygous HAL2 genotypes at two flanking markers (M5-7948 and M5-12422). The values are shown in Mean (SE) with n > 200.

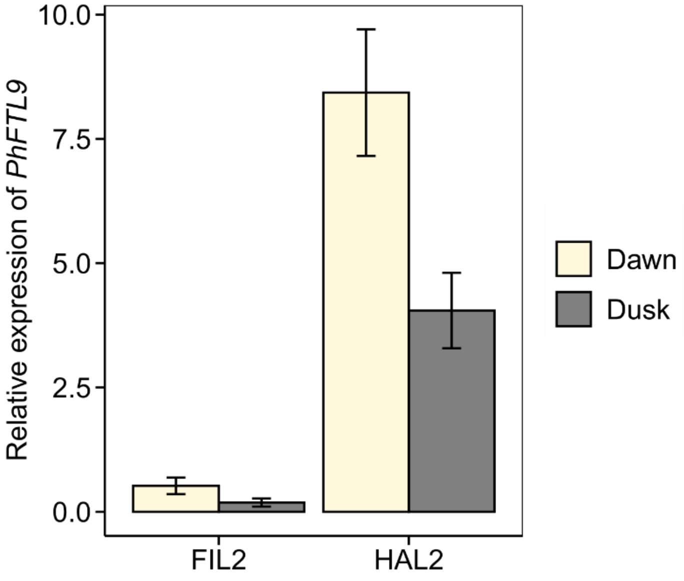

Supplementary Figure S5. Expression analysis of *PhFTL9* gene between HAL2 and FIL2. Leaf samples were harvested two weeks post germination at 08:00 (zeitgeber time 2, cornsilk bars) and 18:00 (zeitgeber time 12, gray bars). Bars and error bars indicate mean values and SE, respectively, based on four biological repeats.

```
Consensus          MXAVEPLVLAHVIRDVLDSFTPTASMRITYNNRLLLAGAELKPSAVVNKPRVDVGGTDLR   60
PhHAL.5G159600     MLAVEPLVLAHVIRDVLDSFTPTASMRITYNNRLLLAGAELKPSAVVNKPRVDVGGTDLR   60
Pahal.5G160000     MSAVEPLVLAHVIRDVLDSFTPTASMRITYNNRLLLAGAELKPSAVVNKPRVDVGGTDLR   60

Consensus          VFYTLVLVDPDAPSPSNPSLREYLHWMVIDIPGTTGASFGQELMFYERPEPRSGIHRMVF  120
PhHAL.5G159600     VFYTLVLVDPDAPSPSNPSLREYLHWMVIDIPGTTGASFGQELMFYERPEPRSGIHRMVF  120
Pahal.5G160000     VFYTLVLVDPDAPSPSNPSLREYLHWMVIDIPGTTGASFGQELMFYERPEPRSGIHRMVF  120

Consensus          VLFRQLGRGTVFAPDMRHNFNCKNFARQYHLDTVAATYFNCQREAGSGGRRFRPESS*    178
PhHAL.5G159600     VLFRQLGRGTVFAPDMRHNFNCKNFARQYHLDTVAATYFNCQREAGSGGRRFRPESS*    178
Pahal.5G160000     VLFRQLGRGTVFAPDMRHNFNCKNFARQYHLDTVAATYFNCQREAGSGGRRFRPESS*    178
```

Supplementary Figure S6. The alignment of PhFTL9 protein sequences between FIL2 (Pahal.5G160000) and HAL2 (PhHAL.5G159600) alleles.

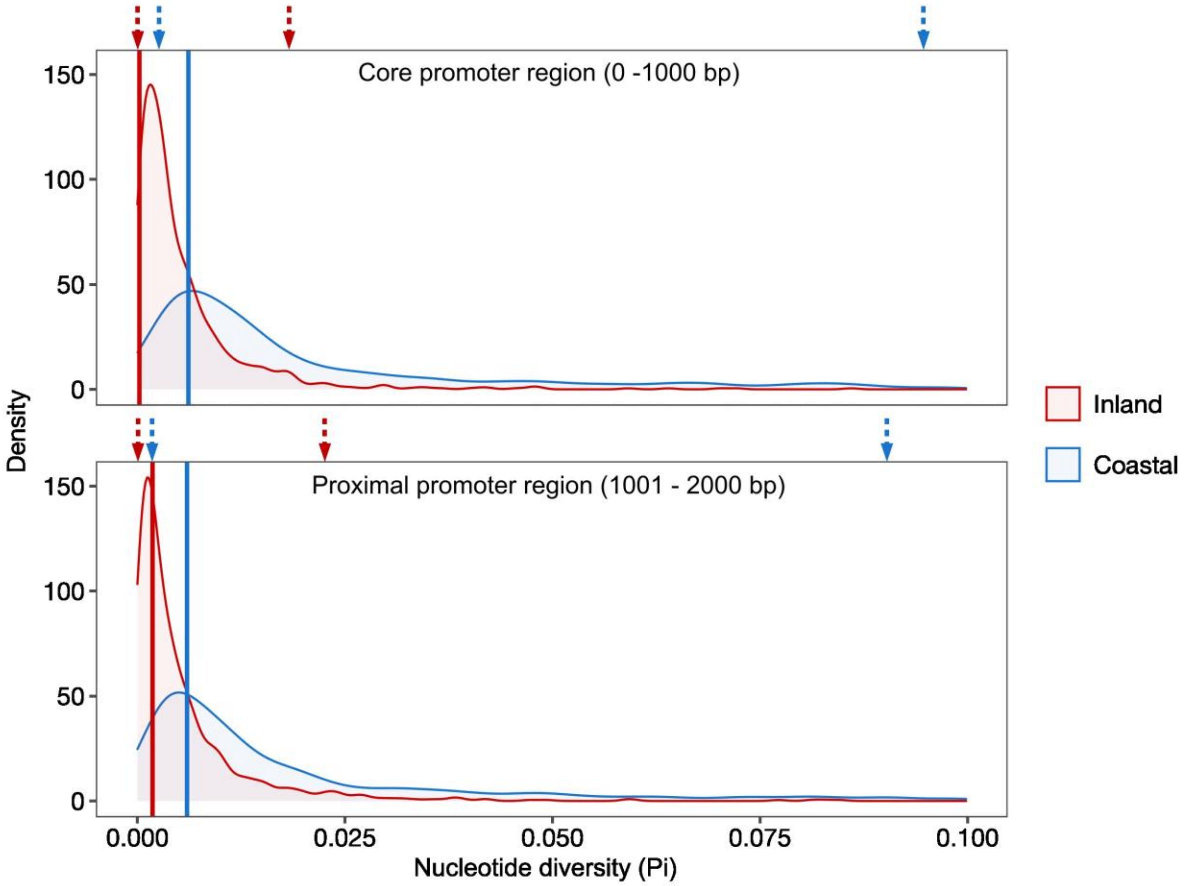

Supplementary Figure S7. The distribution of nucleotide diversity (pi) from the core (left) and proximal (right) promoter regions between inland (red) and coastal (blue) groups. The values of pi from *PhFTL9* promoters were marked by solid red (inland) and blue (coastal) lines. 5% and 95% thresholds were indicated by dashed arrows.

Supplementary Table S1. Performance of flowering time in parents, F_2, and RIL population.

Supplementary Table S2. Directional selection of flowering time divergence using Fraser v-test.

Supplementary Table S3. Primers used in this study.

Supplementary Table S4. QTL-by-environment interaction in RIL population.

Supplementary Table S5. Main effects of QTL at each RIL experiment.

Supplementary Table S6. The pattern of K_a/K_s for proteins and gene expression variation of annotation genes in 380-kb interval of NIL plants.

Supplementary Table S7. ANOVA test for trait differences between parents and NIL plants in the reciprocal transplant experiment.

Supplementary Table S8. Summary of K_a/K_s for *PhFTL9* orthologous pairs in panicoid grasses.
